# Transcriptomic analysis reveals pharmacological mechanisms mediating efficacy of Yangyinghuoxue Decoction in CCl4-induced hepatic fibrosis in rats

**DOI:** 10.3389/fphar.2024.1364023

**Published:** 2024-05-15

**Authors:** Yanming Bai, Shuang Liang, Yanhao Zhou, Bo Zhou

**Affiliations:** ^1^ School of Traditional Chinese Medicine, Ningxia Medical University, Yinchuan, China; ^2^ Yinchuan Hospital of Traditional Chinese Medicine, Ningxia Medical University, Yinchuan, China; ^3^ Ningxia Regional Key Laboratory of Integrated Traditional Chinese and Western Medicine for Prevention and Treatment of High Incidence, Ningxia Medical University, Yinchuan, China

**Keywords:** hepatic fibrosis, Yangyinghuoxue Decoction (YYHXD), transcriptomics, aliphatic acid synthesis and metabolism, AMPK signaling pathway

## Abstract

**Background and purpose:**

As a traditional Chinese medicine formula, Yangyinghuoxue Decoction (YYHXD) is used clinically for therapy of hepatic fibrosis. The pharmacological profile of YYHXD comprises multiple components acting on many targets and pathways, but the pharmacological mechanisms underlying its efficacy have not been thoroughly elucidated. This study aimed at probing the pharmacological mechanisms of YYHXD in the treatment of hepatic fibrosis.

**Methods:**

YYHXD aqueous extract was prepared and quality control using HPLC-MS fingerprint analysis was performed. A CCl_4_-induced rat model of hepatic fibrosis was established, and animals were randomly assigned to six groups: control, low-dose YYHXD (L-YYHXD), medium-dose YYHXD (M-YYHXD), high-dose YYHXD (H-YYHXD), CCl4 model, and colchicine group. Rats in the treatment groups received daily oral administration of YYHXD (5, 10, or 20 g/kg) or colchicine (0.2 mg/kg) for 6 weeks, while the control and model groups received distilled water. Histological analysis, including hematoxylin and eosin (HE) and Masson’s trichrome staining, was performed to evaluate hepatic fibrosis. Serum biochemical markers, such as AST, ALT, HA, and LN, were measured. Inflammatory cytokines (IL-6 and TNF-α) and oxidative stress indicators (SOD, GSH-Px, and MDA) in hepatic tissue were also assessed. Additionally, transcriptomic analysis using RNA-sequencing was conducted to identify differentially expressed genes (DEGs) between the control, CCl4 model, and H-YYHXD groups. Bioinformatics analysis, including differential expression analysis, protein-protein interaction analysis, and functional enrichment analysis, were performed to probe the pharmacological mechanisms of YYHXD. The regulatory effects of YYHXD on fatty acid metabolism and biosynthesis were further confirmed by Oil Red O staining, enzyme activity assays, qPCR, and Western blotting. Western blotting and immunofluorescence staining also validated the involvement of the AMPK signaling pathway in the occurrence and progression of hepatic fibrosis.

**Results:**

HE and Masson’s trichrome staining revealed reduced collagen deposition and improved liver architecture in YYHXD groups compared to the CCl_4_ model group. Serum biochemical markers, including AST, ALT, HA, and LN, were significantly improved in the YYHXD-treated groups compared to the CCl_4_ model group. The levels of inflammatory cytokines (IL-6 and TNF-α) and oxidative stress indicators (decreased SOD and GSH-Px, increased MDA) in hepatic tissue were significantly ameliorated by YYHXD treatment compared to the CCl_4_ model group. Moreover, 96 genes implicated in YYHXD therapy of hepatic fibrosis were screened from the transcriptomic data, which were principally enriched in biological pathways such as fatty acid metabolism and biosynthesis, and the AMPK signaling pathway. Oil Red O staining showed reduced hepatic lipid accumulation by YYHXD in a dose-dependent manner, along with decreased serum TG, TC, and LDL-C levels. Additionally, qPCR and Western blot analyses demonstrated upregulated mRNA and protein expression of key enzymes involved in fatty acid metabolism and biosynthesis, Fasn and Fads2, modulated by YYHXD. YYHXD also dose-dependently enhanced phosphorylation of AMPK as evidenced by Western blotting and immunofluorescence assays.

**Conclusion:**

YYHXD ameliorated CCl_4_-induced hepatic fibrosis in rats through pharmacological mechanisms that involved manifold targets and pathways, including aliphatic acid synthesis and metabolism pathways and the AMPK signaling pathway. This study provided a reference and basis for further research and clinical utilization of YYHXD.

## 1 Introduction

Hepatic fibrosis, a pathological process involving deposition of fibrous connective tissue in the liver, is a consequence of diverse chronic hepatic conditions, such as alcoholic hepatitis, hepatitis B virus infection, hereditary and metabolic liver disorders, and cholestasis (Jangra et al., 2022). It is essentially a manifestation of the body’s self-repair process that occurs over the course of chronic liver injury (Yang et al., 2022). While hepatic fibrosis serves as an integral part of the evolution of chronic hepatic diseases into cirrhosis or hepatocellular carcinoma (HCC), the abnormal tissue structure formed by this repair response is reversible and restorable (Caligiuri et al., 2021). The pathological hallmark of hepatic fibrosis is excessive release and accretion of extracellular matrix (ECM). Activated hepatic stellate cells are responsible for the majority of the ECM release and are critical players in the occurrence of hepatic fibrosis. Currently, there is no FDA-approved anti-fibrotic drug for clinical use, emphasizing the urgent clinical need for anti-fibrotic therapy.

Yangyinghuoxue Decoction (YYHXD) is a traditional Chinese medicine (TCM) formula that has the effects of yangyinghuoxue, promoting qi flow and resolving stasis (Liang et al., 2023). Clinically, YYHXD can be used to treat hepatic fibrosis. Cirrhosis and hepatoma caused by yin deficiency with blood stasis, qi stagnation, and blood stasis (Zhou and Liang, 2015; Liang et al., 2023). YYHXD is a derivative of Siwu Tang, a classic Chinese botanical formula that has been shown to ameliorate hepatic fibrosis by reducing lipid accretion (Chiu et al., 2016), regulating bile acid metabolism (Xue et al., 2021), and modulating immune function (Ma et al., 2022). YYHXD consists of 14 botanical medicines: Danggui, Dihuang, Chuanxiong, Baishao, Chaihu, Chenpi, Chishao, Dahuang, Danshen, Zelan, Huangqin, Zhebeimu, Gancao, and Dazao. According to the principles of traditional Chinese medicine, the combination of these botanical drugs in YYHXD may exert synergistic effects in nourishing blood, promoting blood circulation, resolving blood stasis, regulating qi flow, and related therapeutic actions. Previous studies have demonstrated that YYHXD can ameliorate CCl4-induced hepatic fibrosis in rats (Ma et al., 2018; Liang et al., 2022). However, the pharmacological mechanisms underlying YYHXD therapy of hepatic fibrosis are unclear. Additionally, like other traditional Chinese medicines, understanding of the pharmacological mechanisms of YYHXD is limited by its manifold components and their manifold targets and pathways.

**FIGURE 1:**
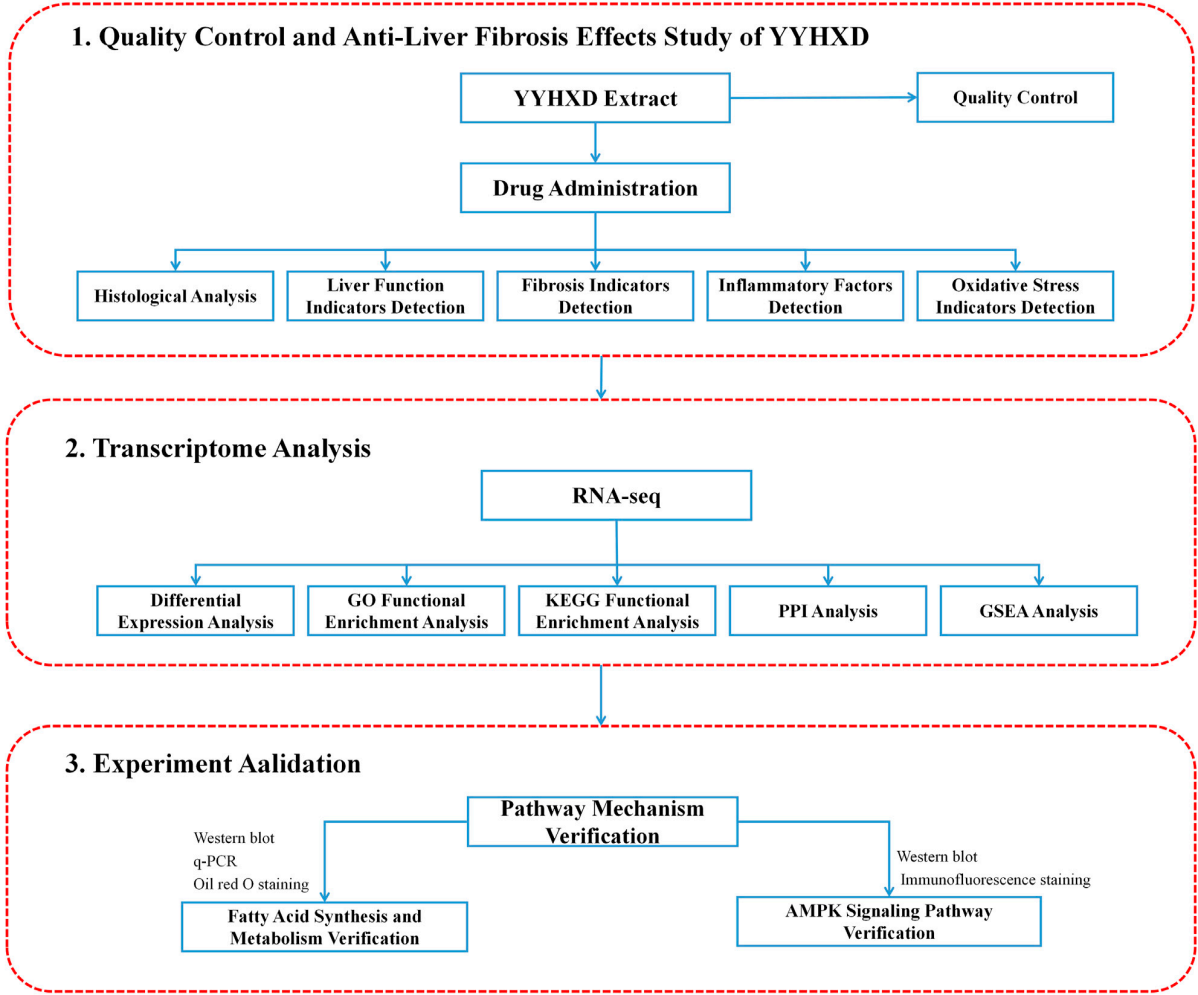
Workflow diagram summary.

Transcriptomics is a technique that is utilized to explore gene expression profiles, which helps to elucidate gene regulation, signaling pathways, and their interactions. Transcriptomics has significant value in TCM pharmacological research, especially when deconvoluting manifold metabolites and targets (Zhang et al., 2021). By quantifying mRNA expression of all genes in cells or tissues, transcriptomics can unravel multiple target effects. Moreover, transcriptomics can build a TCM pharmacological network model, which describes the array of metabolites and targets that are characteristic of TCM, allowing more accurate prediction of TCM efficacy through systems biology and network medicine approaches (Liu et al., 2021). Therefore, in this work, the pharmacological mechanisms underlying YYHXD therapy of hepatic fibrosis were first probed using a transcriptomic approach combined with experimental validation. The study workflow is illustrated in Figure 1.

## 2 Materials and methods

### 2.1 Preparation of YYHXD

YYHXD is a botanical formula consisting of 14 crude drugs in a fixed ratio: Bai-shao, Chai-hu, Chen-pi, Chi-shao, Chuan-xiong, Da-huang, Da-zao, Dan-shen, Dang-gui, Huang-qin, Gan-cao, Di-huang, Ze-lan and Zhe-beimu in a ratio of 4:5:5:5:5:3:3:4:4:4:3:4:3:3 (Table 1). The School of Traditional Chinese Medicine at Ningxia Medical University provided all of the botanical drugs used. The quality of all botanical drugs was corroborated by the School of Pharmacy of Ningxia Medical University. To prepare the medicine, the prescription was crushed and heated twice with eight volumes of water for 1 h each time. The extracts from the two extractions were filtered, combined and concentrated under reduced pressure at 70°C to yield a condensed extract with a concentration of 1.0 g crude drug per mL. This extract was used for quality control and animal administration.

**TABLE 1:** Composition of YYHXD.

Chinese name	English name	Latin name	Application area	Raw botanical drug weight (g)
Bai-shao	White Peony Root	*Paeonia lactiflora* Pall.	Root	12
Chai-hu	Bupleurum Root	*Bupleurum chinense* DC.	Root and rhizome	15
Chen-pi	Tangerine Peel	*Citrus reticulata* Blanco	Fruit peel	15
Chi-shao	Red Peony Root	*Paeonia lactiflora* Pall.	Root	15
Chuan-xiong	Sichuan Lovage Rhizome	*Ligusticum sinense* “Chuanxiong”	Root	15
Da-huang	Rhubarb	*Rheum palmatum* L.	Root and rhizome	9
Da-zao	Chinese Date	*Ziziphus jujuba* Mill.	Fruit	9
Dan-shen	Salvia Root	*Salvia miltiorrhiza* Bunge.	Root and rhizome	12
Dang-gui	Chinese Angelica Root	*Angelica sinensis* (Oliv.) Diels	Root	12
Huang-qin	Baikal Skullcap Root	*Scutellaria baicalensis* Georgi	Root	12
Gan-cao	Licorice Root	*Glycyrrhiza uralensis* Fisch.	Root and rhizome	9
Di-huang	Rehmannia Root	*Rehmannia glutinosa* (Gaert.) Libosch. ex Fisch. et Mey.	Root	12
Ze-lan	Common Bletilla Tuber	*Lycopus* lucidus Turcz. var. hirtus Regel	Tuber	9
Zhe-beimu	Zhejiang Fritillary Bulb	*Fritillaria thunbergii* Miq.	Bulb	9

### 2.2 Quality control of YYHXD

To ensure the quality of YYHXD, a high-performance liquid chromatography-mass spectrometry (HPLC-MS) fingerprint of the final extract was determined. Separation of YYHXD extract by HPLC was performed on a C18 column (100 mm × 2.1 mm, 2.6 µm) (Thermo Fisher Scientific, Wilmington, NC, United States) with a column temperature of 30°C and a flow rate of 1.0 mL/min. The sample injection volume was 0.5 µL. The mobile phase consisted of acetonitrile (A) and water (B). The separation conditions were as follows: 0–10 min, 8% A; 10–20 min, 8%–20% A; 20–30 min, 20%–30% A; 30–35 min, 30%–50% A; 35–40 min, 50%–55% A; 40–50 min, 55%–60% A; and 50–60 min, 90% A. The mass spectrometer was equipped with an electrospray ionization source operating in both positive and negative modes, with spray voltages set at 3,500 and −3,500 V, respectively. The capillary temperature was set at 320°C, and the ion scanning range was 100–1,000 *m/z*. Pure nitrogen gas was used as the carrier gas at a flow rate of 1.0 L/h. Finally, the acquired mass spectral data were compared with those of known reference standards to ensure the quality and consistency of the YYHXD extract.

### 2.3 Animals

Sixty male Wistar rats (200 ± 10 g) were sourced from the Experimental Animal Center of Ningxia Medical University. The ethical protocols for the study were compliant with the Animal Management Regulations provided by the Ministry of Health of the People’s Republic of China, and were ratified by the Ethics Committee of Ningxia Medical University (Approval No. IACUC-NYLAC-2021-009). The rats were housed under controlled environmental conditions (temperature: 22°C–24°C, humidity: 45%–50%, 12-h light/dark cycle), with *ad libitum* access to food and water. Following a 1-week adaptation period, the rats were randomly assigned into six groups (*n* = 10 per group), including a control group, a carbon tetrachloride (CCl4) model group, a low-dose YYHXD group (L-YYHXD), a mid-dose YYHXD group (M-YYHXD), a high-dose YYHXD group (H-YYHXD), and a colchicine group.

### 2.4 Drug administration

For hepatic fibrosis induction, all groups, except the control group, were subjected to subcutaneous injections of CCl_4_ (Sigma-Aldrich, Shanghai, China) diluted with 40% olive oil (Zhang et al., 2013; Fan et al., 2018; Kong et al., 2023). The initial dose of CCl4 was 5 mL/kg, administered for the first week. Subsequently, the rats received 3 mL/kg of CCl_4_ injections twice weekly for the remaining 5 weeks. Starting from the fifth week, the rats in the L-YYHXD, M-YYHXD and H-YYHXD groups received daily oral administration of YYHXD suspension (prepared in distilled water) at doses of 5, 10 and 20 g/10 mL/kg, respectively, for 6 weeks. Rats in the colchicine group received daily oral administration of a colchicine suspension mixed in distilled water at a dose of 0.2 mg/10 mL/kg for 6 weeks. The control and CCl_4_ model groups were orally administered 10 mL/kg of distilled water daily for 6 weeks.

All rats were euthanized 24 h after the final drug treatment using 3% pentobarbital sodium (Servicebio Technology). Blood samples were harvested from the inferior vena cava, and hepatic tissues were acquired.

### 2.5 Histological analysis

To evaluate liver pathology, tissue was fixed in 10% formalin for 24 h, harvested, and then fixed again in 4% paraformaldehyde. Subsequently, the tissue was sliced into 5 µm sections and subjected to hematoxylin and eosin (HE) (Solarbio, Beijing, China) staining to observe histological changes. Masson’s trichrome (Sangon, Shanghai, China) staining was used to assess the degree of hepatic fibrosis. This analysis of hepatic tissue morphology and the extent of fibrosis enabled deeper understanding of the therapeutic effects of YYHXD on hepatic fibrosis.

### 2.6 Serum and hepatic tissue biochemical analysis

Colorimetric assay kits from Shanghai Enzyme Research Biotechnology (Shanghai, China) were utilized to determine the levels of specific serum liver enzymes, including aspartate aminotransferase (AST) and alanine aminotransferase (ALT), according to the manufacturer’s instructions. Enzyme-linked immunosorbent assay (ELISA) kits from Bioswamp (Wuhan, China) were employed to determine serum levels of hyaluronic acid (HA) and laminin (LN) in accordance with the manufacturer’s instructions. Interleukin-6 (IL6) and tumor necrosis factor-alpha (TNF-α) concentrations in hepatic tissue were assessed using ELISA kits from LC-Bio Technologies (Hangzhou, China) following the manufacturer’s instructions. To assess the extent of oxidative stress in each hepatic tissue group, the activities of superoxide dismutase (SOD) and glutathione peroxidase (GSH-Px), together with the level of malondialdehyde (MDA), were determined using an assay kit from Nanjing Jiancheng Biological Engineering Institute (Jiangsu, China) following the provided instructions. Additionally, levels of triglycerides (TG), total cholesterol (TC), and low-density lipoprotein cholesterol (LDL-C) in hepatic tissue were determined using a commercial assay kit from the same supplier in accordance with the manufacturer’s instructions.

### 2.7 RNA extraction and sequencing

Hepatic tissue samples were harvested from nine rats: three from the CCl_4_ model group, three from the control group, and three from the H-YYHXD group. TRIzol Reagent (Life Technologies, Carlsbad, CA, United States) was employed for total RNA extraction from hepatic tissue samples according to the manufacturer’s instructions. RNA purity was ascertained using a NanoDrop 2000 spectrophotometer (Thermo Fisher Scientific). The NEBNextUltra™ RNA library prep kit (New England Biolabs, Ipswich, MA, United States) was used to generate the cDNA library according to the manufacturer’s guidelines. The library quality was determined using an Agilent Bioanalyzer 2100 (Agilent Technologies, Santa Clara, CA, United States). In addition, cluster generation was completed on a cBot Cluster Generation System utilizing TruSeq PE Cluster Kit v4-cBot-HS (Illumina, San Diego, CA, United States) in accordance with the manufacturer’s instructions, followed by library sequencing on an Illumina platform. Clean reads were acquired in accordance with the standard protocols for high-quality sequencing data. Finally, gene expression was quantified using the reads per kilobase per million reads (RPKM) method, which is generally employed for transcriptomic studies to survey gene expression.

### 2.8 RNA sequencing (RNA-seq) data analysis

#### 2.8.1 Gene differential expression analysis

Differential gene expression analysis was conducted using the “limma” R package. Differentially expressed genes (DEGs) in control and CCl_4_ model groups, as well as YYHXD and CCl_4_ model groups, were screened with the threshold of |Fold change| ≥ 2 and *p*-value <0.01. Genes that were significantly elevated in the CCl_4_ model group compared with the control group, and simultaneously significantly suppressed in the YYHXD group compared with the CCl_4_ model group were selected, as well as genes with significantly lower expression in the CCl_4_ model group compared with the control group, and simultaneously significantly elevated expression in the YYHXD group compared with the CCl_4_ model group. These genes were considered to be implicated in the treatment of hepatic fibrosis by YYHXD (therapeutic genes).

#### 2.8.2 Functional enrichment and protein-protein interaction (PPI) analysis

Functional enrichment analysis of the obtained therapeutic genes was conducted by uploading them to the DAVID database (https://david.ncifcrf.gov/). DAVID provides a comprehensive set of functional annotation tools to understand the biological meaning behind large gene lists. The analysis in DAVID included Gene Ontology (GO) term enrichment and Kyoto Encyclopedia of Genes and Genomes (KEGG) pathway enrichment. A *p*-value <0.05 was used as the threshold to identify significantly enriched GO terms and KEGG pathways associated with the therapeutic genes.

Subsequently, Gene Set Enrichment Analysis (GSEA) was performed to identify differential expression of target signaling pathways among the control group, YYHXD group, and CCl_4_ model group using GSEA v4.0.3 software. A normalized enrichment score (NES) > 1 and *p*-value <0.05 were considered significant for differential expression of the signaling pathway.

To further investigate the interactions among the therapeutic genes, a protein-protein interaction (PPI) network analysis was conducted using the STRING database (https://string-db.org/). The therapeutic genes were uploaded to the STRING database, and the resulting PPI network data were imported into Cytoscape software (version 3.7.1) for visualization and topological analysis. Cytoscape was used to identify hub genes within the PPI network based on their ctopological parameters (degree values), which may represent key regulator in the underlying biological processes.

#### 2.8.3 Quantitative reverse transcription-PCR (q-PCR)

The expression levels of fatty acid desaturase 2 (*Fads2*) and fatty acid synthase (*Fasn*) mRNA in hepatic tissue were determined using the q-PCR method. Total RNA extraction from each sample was accomplished using TRIzol. RNA purity and concentration were then determined using a NanoDrop 2000 spectrophotometer (Thermo Fisher Scientific). Subsequently, the RNA extracted from the liver was reverse transcribed into cDNA using the PrimeScript RT Master Mix kit (Takara Bio, Kusatsu, Japan). Primers for fatty acid *Fads2*, *Fasn* and *β-actin* were designed using Primer Premier 5.0 software. *β-actin* was used as the internal reference gene. q-PCR was implemented on a CFX Connect machine (BIO-RAD, Hercules, CA, United States) using SYBR Green PCR Mix (Monad Biotech, Wuhan, China). The primer sequences utilized in the q-PCR are shown in Table 2.

**TABLE 2:** Primer sequences for q-PCR.

Gene	Forward primer (5ʹ–3ʹ)	Reverse primer (5ʹ–3ʹ)	Product size (bp)
*Fads2*	ATT​CGG​GAG​AAG​ATG​CTA​CG	CCC​TGA​AGT​CCT​CGG​TGA​T	156
*Fasn*	TCG​CAT​GAA​CAC​TCT​GGA​GAT​G	TCT​GTG​AGG​GAC​TCT​GGT​CTT​TG	146
*β-actin*	AGA​TTA​CTG​CCC​TGG​CTC​CTA​G	CAT​CGT​ACT​CCT​GCT​TGC​TGA​T	144

### 2.9 Western blotting

Western blot analysis was used to determine the protein expression levels of FASN, FADS2, peroxisome proliferator-activated receptor-alpha (PPARα), AMPK, and p-AMPK in hepatic tissue. Briefly, appropriate amounts of frozen hepatic tissue were ground in liquid nitrogen, and total protein extraction was completed using RIPA lysis buffer mixed with phosphate inhibitor. Protein concentrations were then determined using the BCA protein assay reagent (Abcam, Cambridge, United Kingdom). Protein samples were denatured by heating for 10 min and then separated by 10% sodium dodecyl-sulfate polyacrylamide gel electrophoresis (SDS-PAGE). Subsequently, the samples were transferred onto polyvinylidene difluoride (PVDF) membranes on an electrophoresis system. Blocking of the PVDF membranes was accomplished at room temperature during 1 h using 5% skim milk. Primary antibodies, including FASN (1:1,000), FADS2 (1:1,000), PPARα (1:1,000), AMPK (1:1,000), p-AMPK (1:1,000) and β-actin (1:1,000) (Elabscience Biotechnology, Wuhan, China) were added and incubated overnight at 4°C. The membranes were then incubated with a fluorescent secondary antibody (1:5,000) (Elabscience Biotechnology) for 1 h at room temperature. The membranes were developed by enhanced chemiluminescence (ECL) and exposed in a gel imager. The relative gray value of the target protein was assessed using Image J software. β-Actin acted as a loading control.

### 2.10 Immunofluorescence staining

Immunofluorescence staining was used to analyze the phosphorylation level of AMPK in hepatic tissues from CCl_4_ model rats treated with YYHXD. Specifically, paraffin-embedded hepatic tissues were sliced into 4 µm sections, and then dewaxed and rehydrated. Endogenous peroxidase activity was quenched by incubating the sections in 3% hydrogen peroxide for 10 min. Section blocking was achieved using 5% bovine serum albumin (BSA) solution for 30 min, which was followed by incubation with primary antibody against p-AMPK (1:200) (Abcam) at 4°C overnight. After washing with phosphate-buffered saline (PBS), sections were incubated with secondary antibody (1:1,000) (Elabscience Biotechnology) at room temperature for 30 min, followed by counterstaining with 4,6-diamidino-2-phenylindole (DAPI) (Thermo Fisher Scientific). Fluorescence images were acquired using a fluorescence microscope (Nikon Corporation, Tokyo, Japan).

## 3 Results

### 3.1 Quality control of YYHXD

**FIGURE 2:**
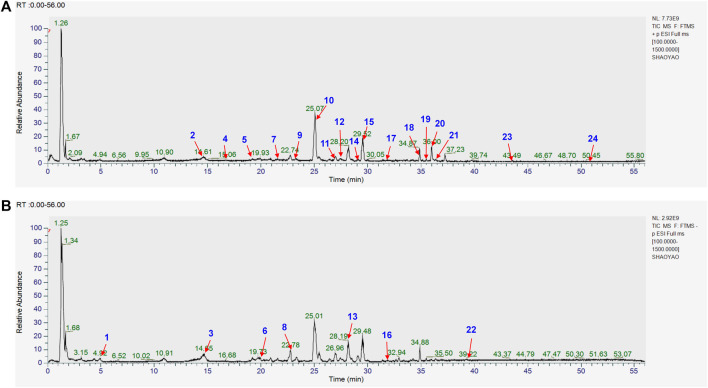
Total ion chromatogram of YYHXD based on HPLC-MS analysis. **(A)** Positive ion mode. **(B)** Negative ion mode.

The chemical composition of YYHXD was assayed by HPLC-MS and a characteristic fingerprint was constructed. The fingerprint contained 24 metabolites (shown in Figure 2), including 17 metabolites identified in positive ion mode: paeoniflorin, isoferulic acid, isoliquiritigenin, naringenin chalcone, senkyunolide H, baicalin, apigenin 7-O-glucuronide, 6-O-methylscutellarin, emodin, oroxindin, aloe-emodin, tangeritin, nobiletin, wogonin, 5,2′-dihydroxy-6,7,8,6′-tetramethoxyflavone, cryptotanshinone, and tanshinone IIA; and 7 metabolites detected in negative ion mode: catechin, albiflorin, rutin, neohesperidin, chrysin, luteolin, and apigenin. The characteristic chemical composition of YYHXD is shown in Table 3.

**TABLE 3:** Identification of characteristic components in YYHXD.

ID	Name	Formula	RT [min]	Calc. MW	m/z	Reference ion	mz/mz	Structure
**1**	Catechin	C_15_H_14_O_6_	4.935	290.07952	289.07205	[M-H]^−^	335.07767([M+FA-H]-), 245.08189, 221.08162, 205.05019, 123.04431, 109.02861	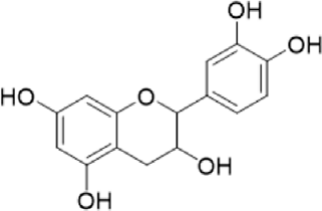
**2**	Paeoniflorin	C_23_H_28_O_11_	14.622	497.19109	498.19836	[M+H]^+^	197.08177, 179.07076, 151.07571, 133.06520, 105.07053	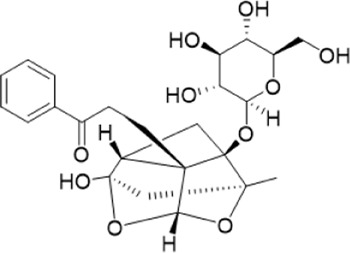
**3**	Albiflorin	C_23_H_28_O_11_	14.645	480.16325	525.16144	[M+FA-H]^−^	362.26999, 327.10812, 280.79984, 220.24370, 121.02087	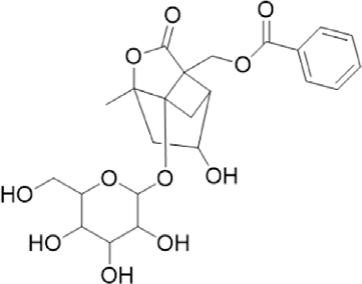
**4**	Isoferulic acid	C_10_H_10_O_4_	16.676	194.05852	195.0658	[M+H]^+^	177.05501, 145.02875, 117.03398	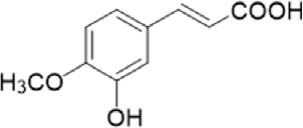
**5**	Isoliquiritigenin	C_15_H_12_O_4_	19.213	256.07417	257.08145	[M+H]^+^	239.07040, 211.07571, 147.04439, 137.02367, 119.04963	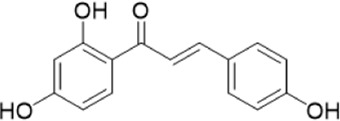
**6**	Rutin	C_27_H_30_O_16_	19.751	564.14818	609.14636	[M+FA-H]^−^	300.02774, 271.02499, 255.02986, 227.03465, 151.00288, 108.02105	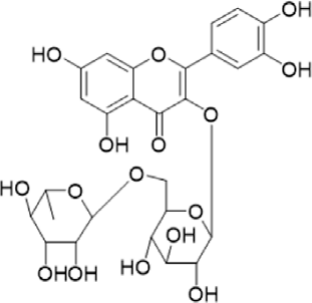
**7**	Naringeninchalcone	C_15_H_12_O_5_	21.554	272.06913	273.07645	[M+H]^+^	171.02922, 153.01859, 147.04442, 119.01889, 107.04961	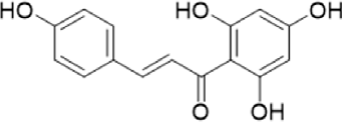
**8**	Neohesperidin	C_28_H_34_O_15_	22.754	610.19037	609.18262	[M-H]^−^	343.08179, 325.07324, 301.07199, 286.04849, 196.00092, 174.03175, 125.02368	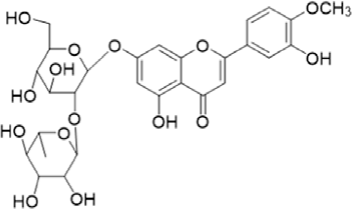
**9**	Senkyunolide H	C_12_H_16_O_4_	23.029	206.09503	207.10231	[M+H]^+^	189.09155, 165.05524, 119.08614, 105.07050	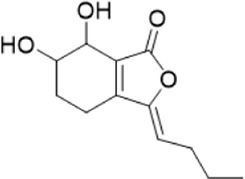
**10**	Baicalin	C_21_ H_18_O_11_	25.064	446.0856	447.09308	[M+H]^+^	469.07315 ([M+Na]), 423.79083, 271.06061, 225.05571, 105.03415	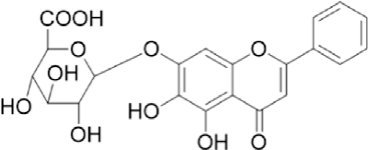
**11**	Apigenin 7-O-glucuronide	C_21_H_18_O_11_	26.979	446.08562	447.09323	[M+H]^+^	395.81219, 370.43393, 271.06073, 237.06723, 225.05511, 180.41391, 169.01369, 105.03422	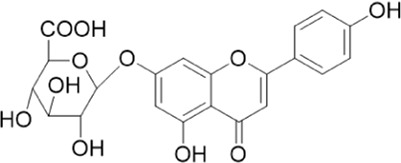
**12**	6-O-Methylscutellarin	C_22_H_20_O_12_	27.448	476.09655	477.10425	[M+H]^+^	499.08658([M+Na]), 396.56082, 301.07132, 286.04779, 202.01146, 184.00076, 156.00566, 115.08618	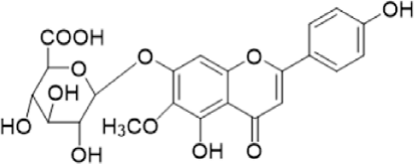
**13**	Chrysin	C_15_H_10_O_4_	28.189	254.05781	253.05045	[M-H]^−^	225.05571, 210.03284, 181.06470, 115.70124	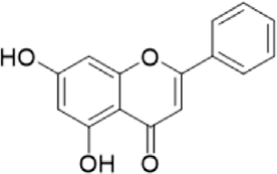
**14**	Emodin	C_15_H_10_O_5_	29.032	270.05325	271.06076	[M+H]^+^	229.04991, 225.05487, 201.05585, 197.06047, 169.06615	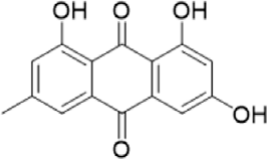
**15**	Oroxindin	C_22_H_20_O_11_	29.508	460.10118	461.10867	[M+H]^+^	483.09113 ([M+Na]), 285.07642, 270.05286, 144.05705	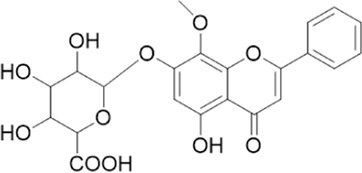
**16**	Luteolin	C_15_H_10_O_6_	31.788	286.04817	285.04089	[M-H]^−^	255.03049, 227.03520, 213.05630, 143.04944, 109.02866	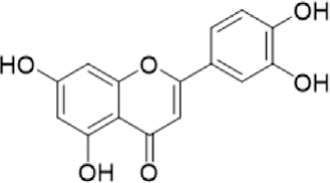
**17**	Aloe-emodin	C_15_H_10_O_5_	31.985	270.05364	271.06091	[M+H]^+^	253.05025, 241.05038, 225.05519, 197.05988, 169.06531, 141.06972	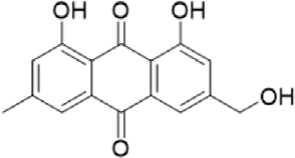
**18**	Tangeritin	C_20_H_20_O_7_	34.856	372.12175	373.12903	[M+H]^+^	343.082, 329.10251, 315.08722, 296.06866, 254.05923, 181.01445, 151.03897	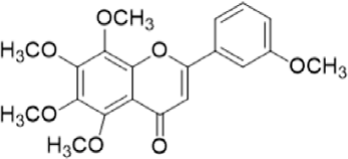
**19**	Nobiletin	C_21_H_22_O_8_	35.584	402.13278	403.14005	[M+H]^+^	425.12191([M+Na]+), 369.09665, 355.08261, 345.06381, 284.06857	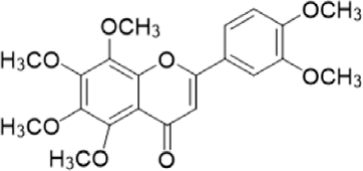
**20**	Wogonin	C_16_H_12_O_5_	35.952	284.06892	285.07635	[M+H]^+^	269.04425, 242.05856, 197.05891, 116.06232	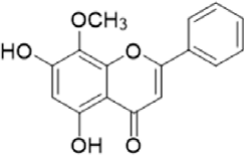
**21**	5,2′-Dihydroxy-6,7,8,6′-tetramethoxyflavone	C_19_H_18_O_8_	36.374	374.10105	375.10852	[M+H]^+^	327.05078, 302.04294, 271.06088, 227.05565, 215.01918, 149.06023, 121.06543	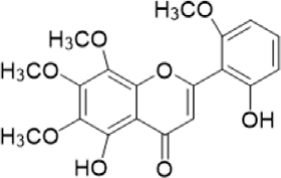
**22**	Apigenin	C_15_H_10_O_5_	39.244	270.05314	269.04587	[M-H]^−^	241.05096, 225.05565, 181.06563, 135.66243, 120.49317	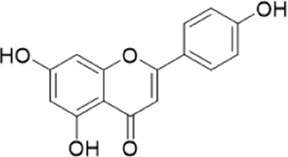
**23**	Cryptotanshinone	C_19_H_20_O_3_	43.396	296.14206	297.14932	[M+H]^+^	319.13132([M+Na]+), 268.11017, 261.12799, 254.09451, 221.09726, 207.08147, 181.10123, 116.64880	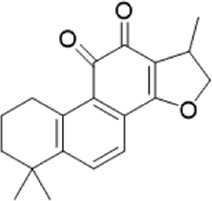
**24**	Tanshinone IIA	C_19_H_18_O_3_	50.732	294.12669	295.13394	[M+H]^+^	277.12308, 252.07915, 235.07915, 207.08168, 192.09383, 111.03707	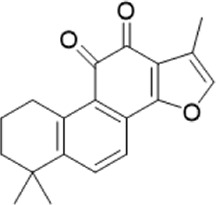

The bold font (ID) numbers correspond to the IDs of each feature peak in Figure 2.

### 3.2 YYHXD alleviated histopathological changes in hepatic fibrosis rats

**FIGURE 3:**
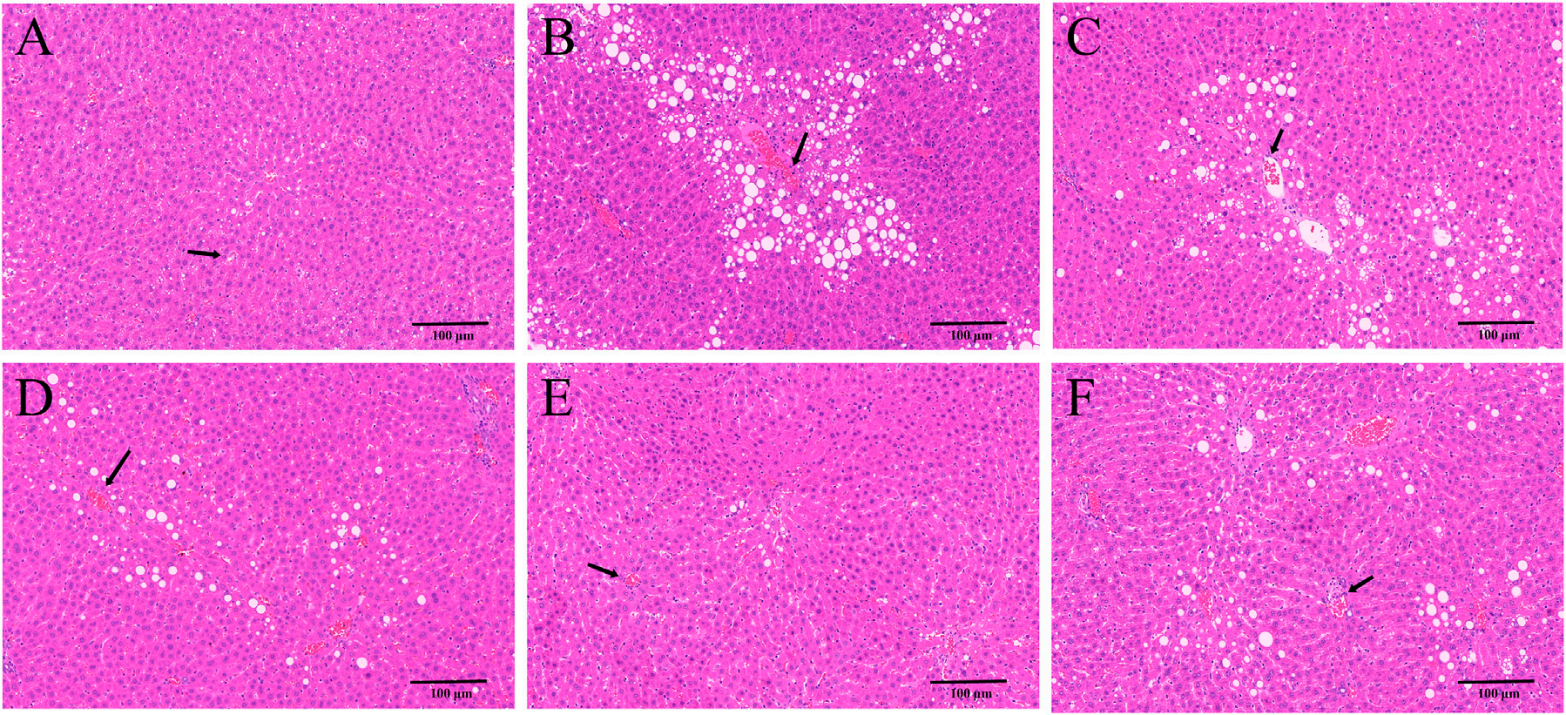
HE staining. HE staining in control **(A)**, CCl_4_ model **(B)**, L-YYHXD **(C)**, M-YYHXD **(D)**, H-YYHXD **(E)**, and colchicine **(F)** groups.

**FIGURE 4:**
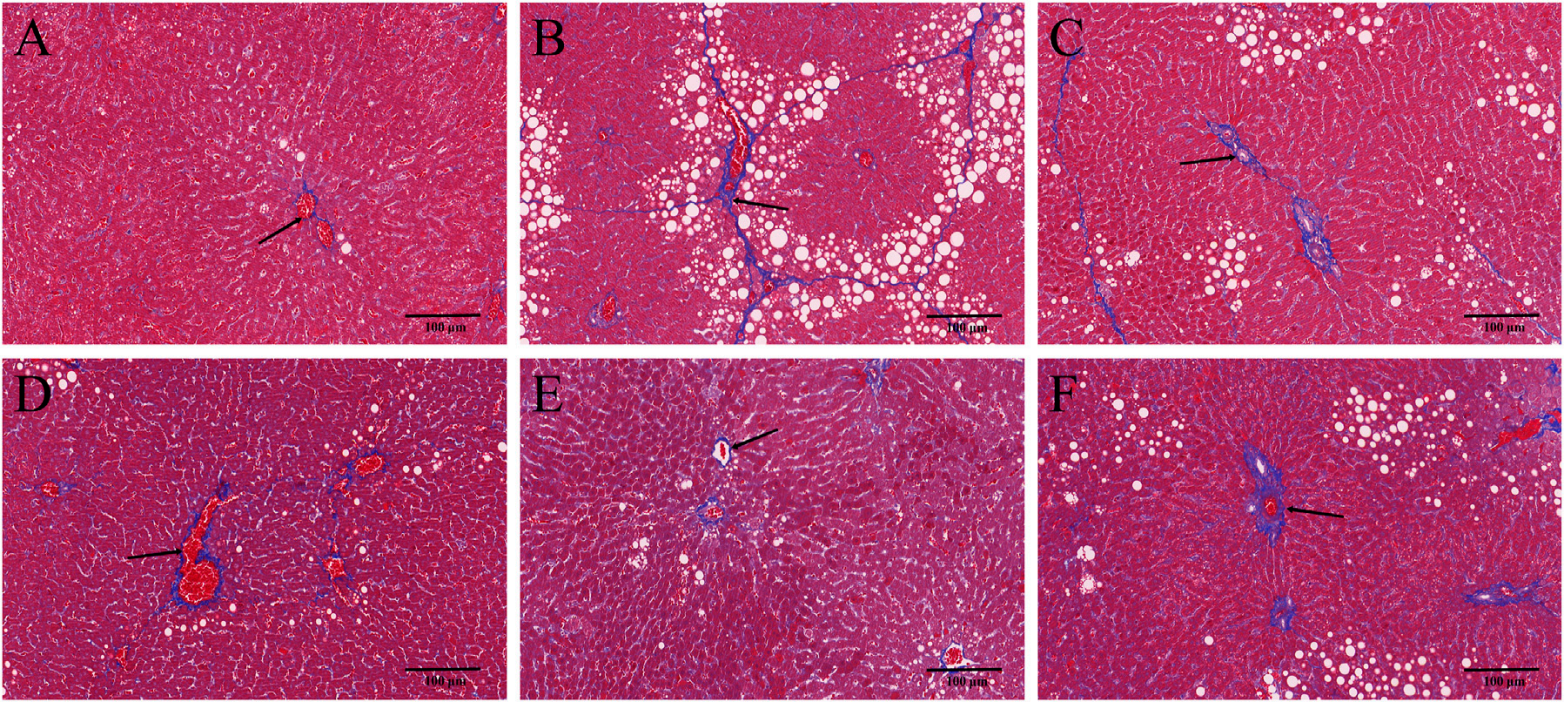
Masson staining. Masson staining in control **(A)**, CCl_4_ model **(B)**, L-YYHXD **(C)**, M-YYHXD **(D)**, H-YYHXD **(E)** and colchicine **(F)** groups.

HE and Masson staining of rat hepatic tissues were used to probe the effects of YYHXD on pathological changes in rats with hepatic fibrosis. The results are shown in Figures 3, 4. Hepatic tissues in the control group exhibited normal lobule and cellular structures, with no inflammatory cell infiltration or fibrous tissue proliferation between the hepatic lobules. In the CCl_4_ model group, however, the hepatic tissue showed obvious steatosis and vacuolization, disorganized hepatic lobule structure, disorganized arrangement of hepatocyte cords, massive deposition of collagen fibers, and obvious infiltration of inflammatory cells. Compared with the CCl_4_ model group, the severity of CCl_4_-induced hepatic pathological changes was remarkably attenuated in the YYHXD and positive control groups. Only a small amount of collagen fiber deposition was apparent in the YYHXD high dose group. The results suggested that YYHXD ameliorated CCl_4_-induced hepatic injury and fibrosis in rats.

### 3.3 YYHXD ameliorated serum and hepatic tissue indicators in hepatic fibrosis rats

**FIGURE 5:**
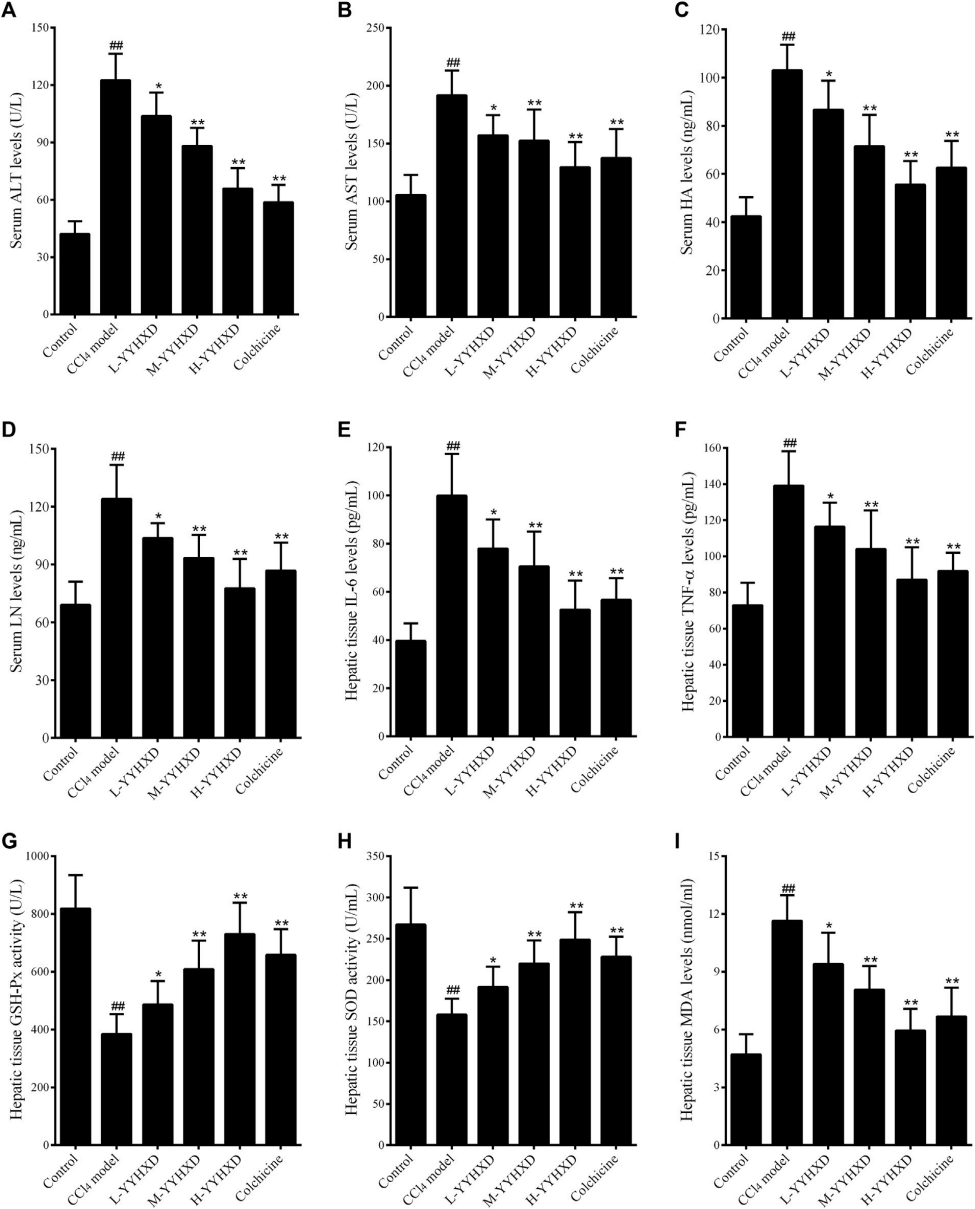
Effect of YYHXD on serum and hepatic tissue indicators in hepatic fibrosis rats. **(A)** ALT; **(B)** AST; **(C)** HA; **(D)** LN; **(E)** IL6; **(F)** TNF-α; **(G)** GSH-Px; **(H)** SOD; **(I)** MDA. ^##^ *p*-value <0.01, compared with the control group. **p-value <0.01, compared with the CCl_4_ model group.

In this work, serum ALT and AST concentrations were determined to assess hepatic function. After CCl_4_ administration, serum levels of ALT and AST were significantly elevated, implying that CCl_4_ induced liver dysfunction in rats. YYHXD significantly reduced the elevated serum ALT and AST induced by CCl_4_ in a dose-dependent manner, indicating that YYHXD restored liver function in rats with hepatic fibrosis (Figures 5A, B).

To further assess the anti-fibrotic effect of YYHXD, serum concentrations of HA and LN were determined by ELISA. The results showed that, compared with the control group, the CCl_4_ model group displayed significantly elevated serum levels of HA and LN. However, concentrations of these indicators were notably lower in the YYHXD and positive control groups compared with the CCl_4_ model group, indicating that YYHXD and colchicine have anti-fibrotic effects. It is worth noting that the serum concentrations of HA and LN were lower in the high-dose YYHXD group compared with those in the colchicine group (Figures 5C, D).

To assess the effects of YYHXD on immune damage in hepatic fibrosis, levels of inflammatory factors IL6 and TNF-α in hepatic tissue were also determined. The results, as shown in Figures 3E, F, demonstrated that both YYHXD and colchicine significantly reduced the elevated concentrations of IL6 and TNF-α in the hepatic tissue of CCl_4_ model rats. The antiphlogistic effect of YYHXD increased at higher doses.

Analysis of oxidative stress indicators revealed that, compared with the control group, concentrations of GSH-Px and SOD in hepatic tissue fell significantly, while the concentration of MDA increased in the CCl_4_ model group. YYHXD significantly and dose-dependently ameliorated oxidative stress damage in hepatic tissue of rats with hepatic fibrosis by upregulating levels of GSH-Px and SOD, and downregulating the concentration of MDA in hepatic tissue. The antioxidant effect of high-dose YYHXD (20 g/10 mL/kg) was superior to that of colchicine (0.2 mg/10 mL/kg) (Figures 5G–I).

### 3.4 Analysis of RNA-seq data

#### 3.4.1 Screening of genes implicated in YYHXD therapy of hepatic fibrosis

**FIGURE 6:**
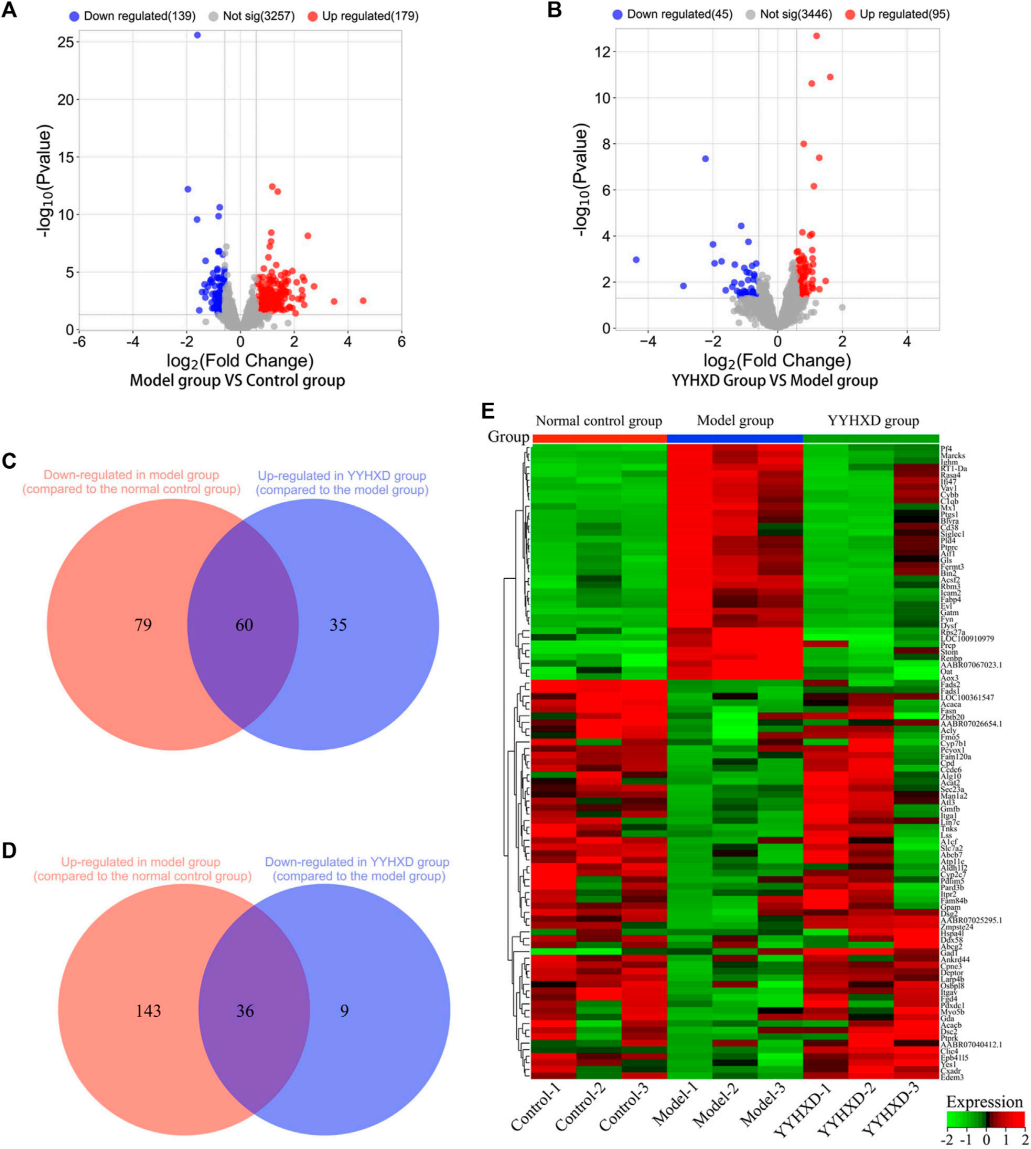
Screening of genes implicated in YYHXD therapy for hepatic fibrosis. **(A,B)** Volcano plots for differential analyses between the CCl_4_ model and control groups, and between the YYHXD and CCl_4_ model groups; **(C)** Therapeutic genes identified by comparing gene expression differences between the CCl_4_ model and control groups, as well as between the CCl_4_ model and YYHXD groups. Certain genes were observably upregulated in the CCl_4_ model group but significantly downregulated in the YYHXD group. **(D)** Therapeutic gene screening. These genes were notably downregulated in the CCl_4_ model group but significantly upregulated in the YYHXD group. **(E)** Expression heat map of therapeutic genes.

Using transcriptome sequencing technology, gene expression in rat hepatic tissues from the control, YYHXD, and CCl_4_ model groups was used to probe the pharmacological mechanism of YYHXD in the treatment of hepatic fibrosis. A threshold of |fold-change| ≥ 1.5 and adjusted *p*-value <0.01 was set. A total of 318 differentially expressed genes were identified between the control and CCl_4_ model groups, including 179 that were upregulated and 139 that were downregulated (Figure 6A). Moreover, 140 differentially expressed genes were discovered between the CCl_4_ model and YYHXD groups, including 95 that were upregulated and 45 that were downregulated (Figure 6B). By comparing these differentially expressed genes, 96 genes implicated in the treatment of hepatic fibrosis by YYHXD (therapeutic genes) were identified. Expression of these therapeutic genes was significantly altered by CCl_4_ treatment, and the changes were significantly reversed by YYHXD. The changes included 23 genes that were significantly elevated in the CCl_4_ model group compared with the control group, but significantly reduced in the YYHXD group compared with the CCl_4_ model group (Figure 6C), as well as 35 genes that were significantly suppressed in the CCl_4_ model group compared with the control group, but significantly elevated in the YYHXD group compared with the CCl_4_ model group (Figure 6D). The expression heat map of these therapeutic genes is shown in Figure 5E. Of note, *Fasn* and *Fads2* were two genes that exhibited the largest differences among the control, YYHXD, and CCl_4_ model groups (CCl_4_ model group vs. control group: Fold change >2, *p*-value <0.05; YYHXD group vs. CCl_4_ model group: Fold change >2, *p*-value <0.05), suggesting that *Fasn* and *Fads2* may be key players in hepatic fibrosis and in therapy with YYHXD.

#### 3.4.2 Functional enrichment and PPI analysis

**FIGURE 7:**
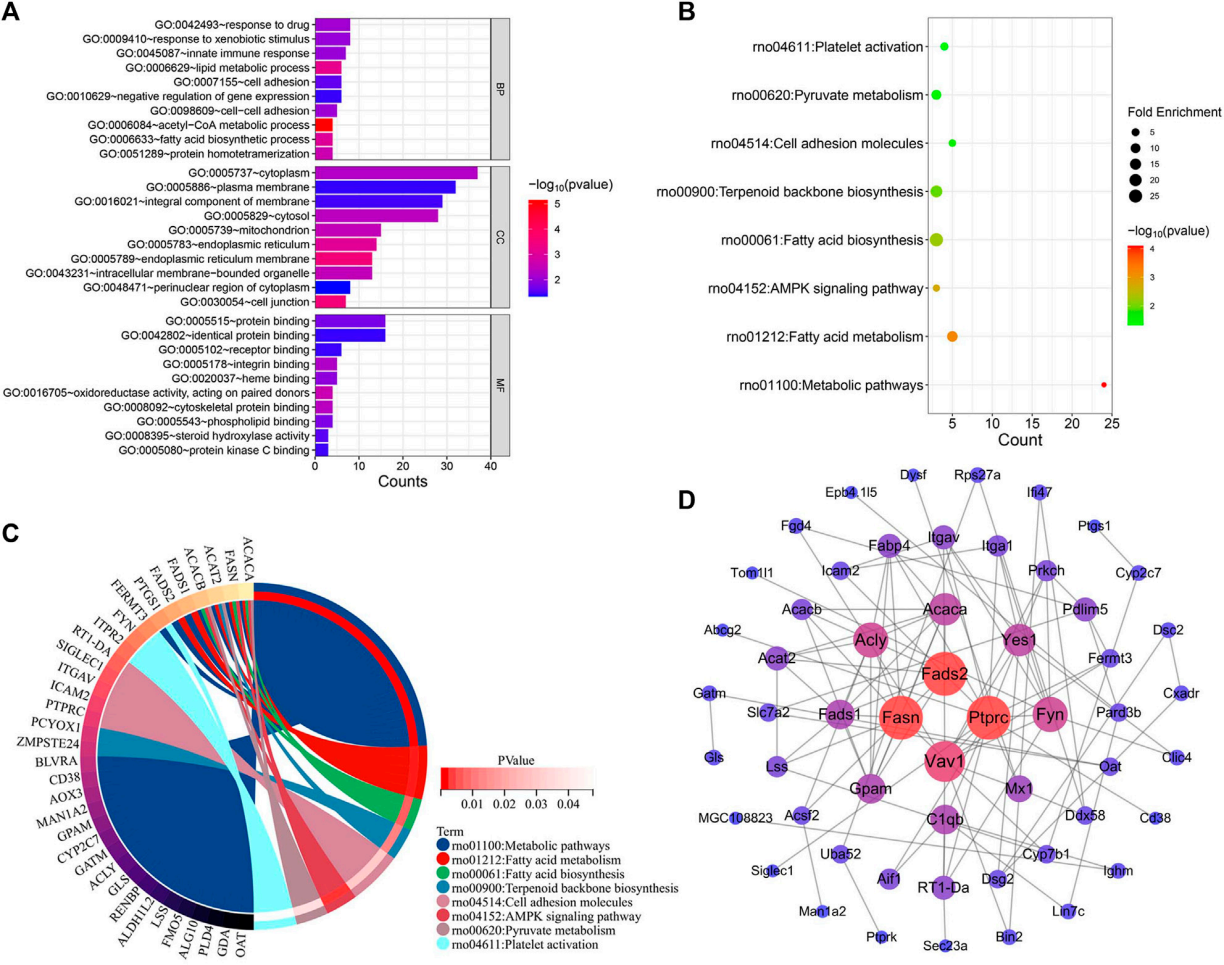
Functional enrichment and PPI analysis of therapeutic genes. **(A)** GO annotation. **(B)** KEGG pathway enrichment analysis. **(C)** Interaction of KEGG signaling pathways and their associated therapeutic genes. **(D)** PPI analysis.

The genes implicated in YYHXD therapy of hepatic fibrosis were uploaded onto the DAVID database to conduct GO and KEGG pathway enrichment analysis to probe the possible biological pathways involved in hepatic fibrosis treatment by YYHXD. The GO annotation results revealed that the therapeutic genes were significantly enriched in 30 biological processes (BP), 20 cellular components (CC), and 17 molecular functions (MF) (*p*-value <0.05). The BP included response to drug, response to xenobiotic stimulus, innate immune response, lipid metabolism and cell adhesion. The CC terms included cytoplasm, plasma membrane, cytosol, mitochondrion, and integral component of membrane. In terms of MF, the therapeutic genes were enriched in protein binding, identical protein binding, receptor binding, integrin binding, and heme binding predominantly. The top 10 significantly enriched GO terms in BP, CC, and MF based on the count ranking are shown in Figure 7A. Moreover, a total of eight KEGG pathways were identified after removing signaling pathways that were implicated in the disease (Figures 7B, C). The obtained KEGG pathways were metabolic pathways, aliphatic acid metabolism, aliphatic acid biosynthesis, terpenoid backbone biosynthesis, cell adhesion molecules, AMPK signaling pathway, pyruvate metabolism and platelet activation.

**FIGURE 8:**
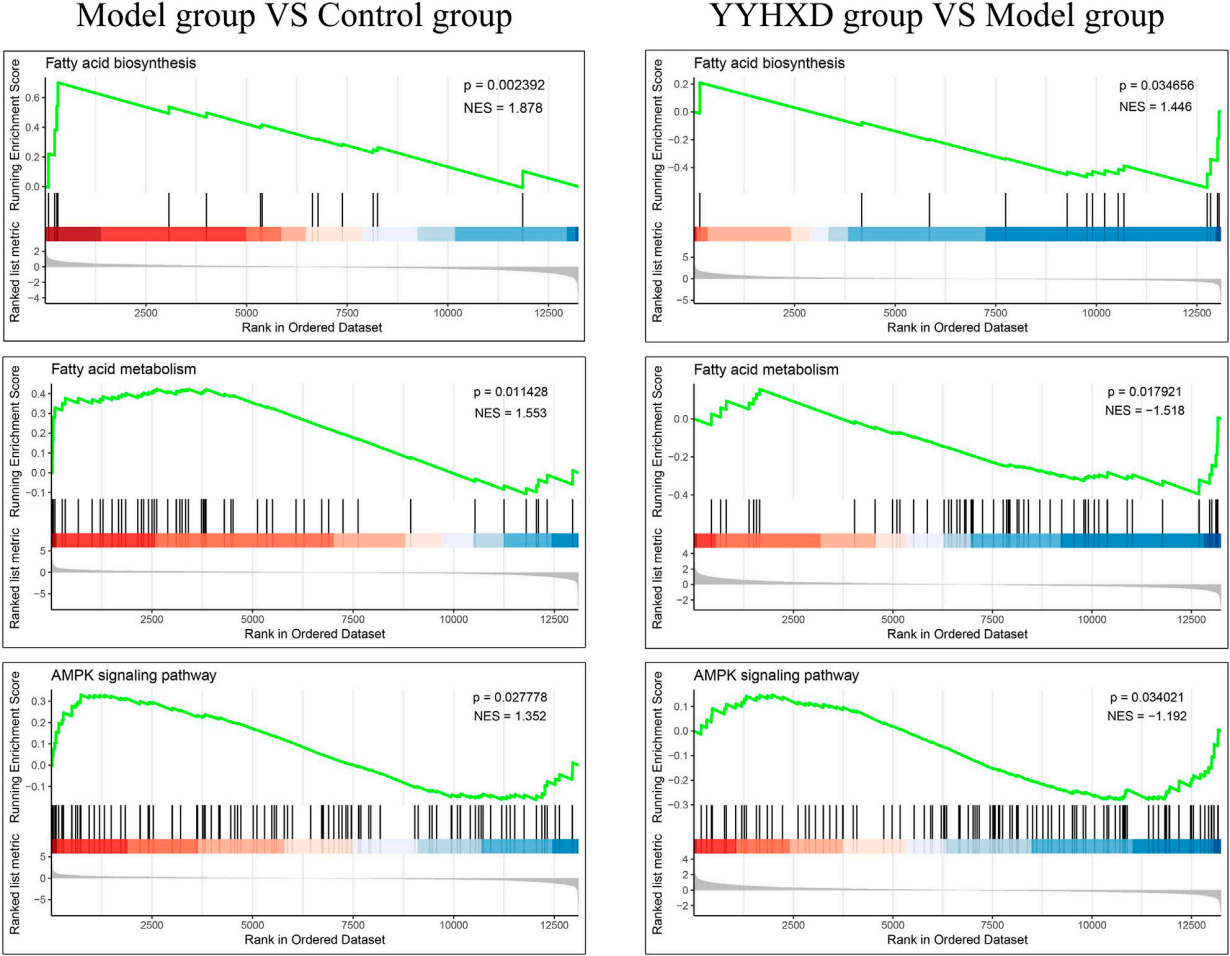
GSEA results for aliphatic acid biosynthesis, aliphatic acid metabolism, and the AMPK signaling pathway.

Additionally, we confirmed the expression differences of these eight KEGG signaling pathways in the CCl_4_ model, YYHXD, and control groups by Gene Set Enrichment Analysis (GSEA). The results are presented in Table 4. Among the eight pathways, seven were found to be regulated by YYHXD through GSEA. Notably, two pathways, cell adhesion molecules and platelet activation, were markedly downregulated (NES <0, *p*-value <0.05) in the CCl_4_ model group compared with the control group, but were upregulated (NES >0, *p*-value <0.05) in the YYHXD group compared with the CCl_4_ model group. On the other hand, five pathways, including aliphatic acid biosynthesis, aliphatic acid metabolism, terpenoid backbone biosynthesis, AMPK signaling pathway, and pyruvate metabolism, were prominently upregulated (NES >0, *p*-value <0.05) in the CCl_4_ model group compared with the control group, but displayed significant downregulation (NES <0, *p*-value <0.05) in the YYHXD group compared with the CCl_4_ model group. The GSEA results for aliphatic acid biosynthesis, aliphatic acid metabolism, and the AMPK signaling pathway are shown in Figure 8. Overall, these discoveries provide valuable insight into the mechanisms underlying the therapeutic effects of YYHXD on hepatic fibrosis, suggesting regulation of aliphatic acid metabolism through modulation of the AMPK signaling pathway.

**TABLE 4:** Seven KEGG signaling pathways identified by GSEA.

ID	Description	CCl_4_ model group vs. Control group		YYHXD group vs. CCl_4_ model group	
		NES	*p*-value	NES	*p*-value
ko04611	Platelet activation	−1.902	0.001107	1.765	0.001236
ko04514	Cell adhesion molecules	−1.876	0.001104	1.844	0.001233
ko04152	AMPK signaling pathway	1.352	0.027778	−1.192	0.034021
ko01212	Aliphatic acid metabolism	1.553	0.011428	−1.518	0.017921
ko00900	Terpenoid backbone biosynthesis	1.613	0.011952	−1.804	0.008876
ko00620	Pyruvate metabolism	1.445	0.044554	−1.489	0.026756
ko00061	Aliphatic acid biosynthesis	1.878	0.002392	−1.446	0.034656

The interactions between these 96 therapeutic genes are shown in the PPI network diagram (Figure 7D). *Fasn* and *Fads2* were two of the most significant genes in the PPI network in terms of topological parameters (degree values). The results suggested that FASN and FADS2 may function as pivotal players in the treatment of hepatic fibrosis by YYHXD.

### 3.5 YYHXD regulated aliphatic acid synthesis and metabolism in hepatic fibrosis rats

**FIGURE 9:**
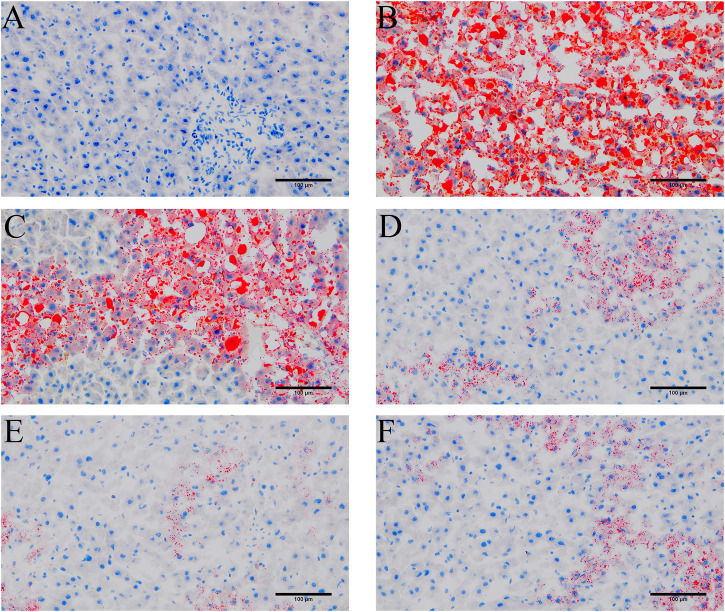
Oil Red O staining. Oil Red O staining in control **(A)**, CCl_4_ model **(B)**. L-YYHXD **(C)**, M-YYHXD **(D)**, H-YYHXD **(E)** and colchicine **(F)** groups.

**FIGURE 10:**
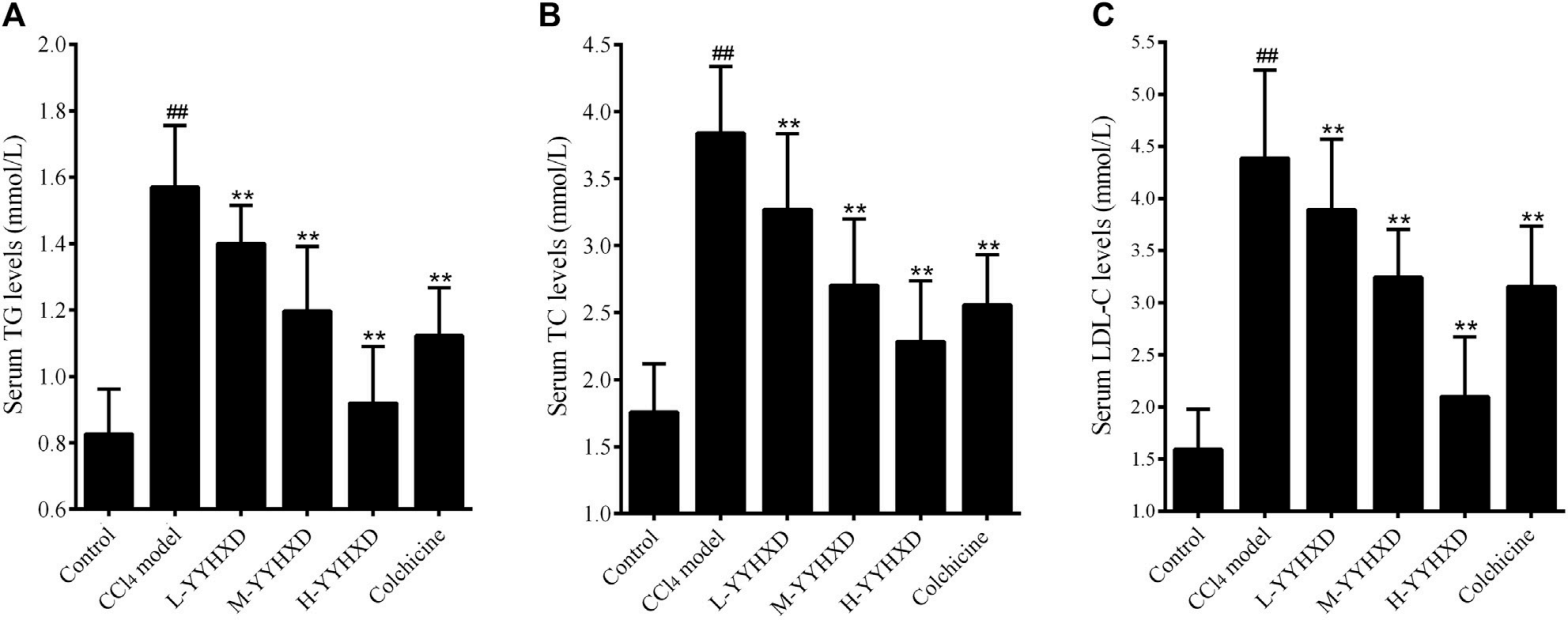
Effect of YYHXD on serum indicators of aliphatic acid synthesis and metabolism in hepatic fibrosis rats. **(A)** TG. **(B)** TC. **(C)** LDL-C. ^##^ *p*-value <0.01, compared with the control group. **p-value <0.01, compared with the CCl_4_ model group.

**FIGURE 11:**
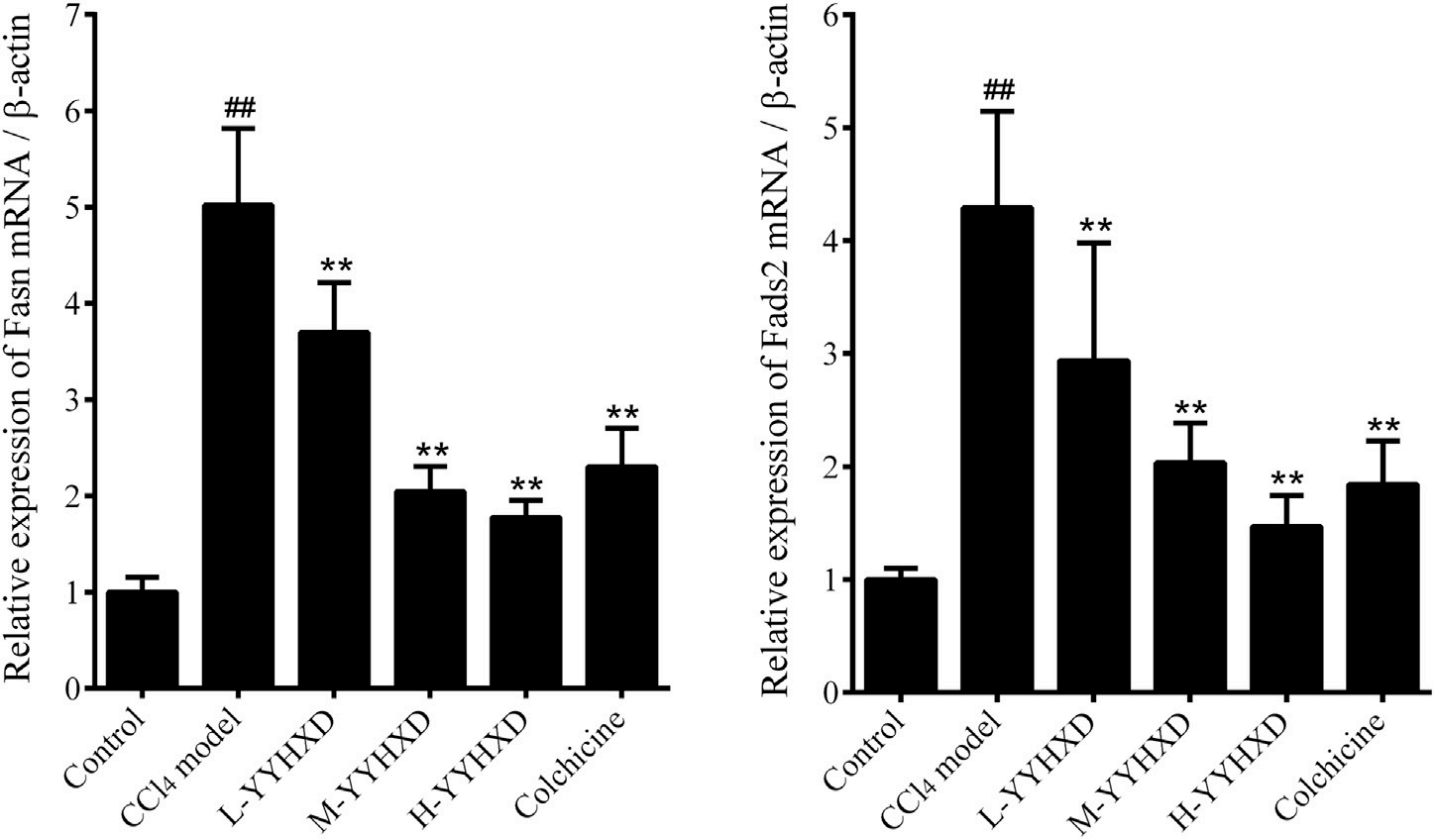
Effect of YYHXD on expression of *Fasn* and *Fads2* mRNA in hepatic fibrosis rats. ^##^ *p*-value <0.01, compared with the control group. ^**^ *p*-value <0.01, compared with the CCl_4_ model group.

**FIGURE 12:**
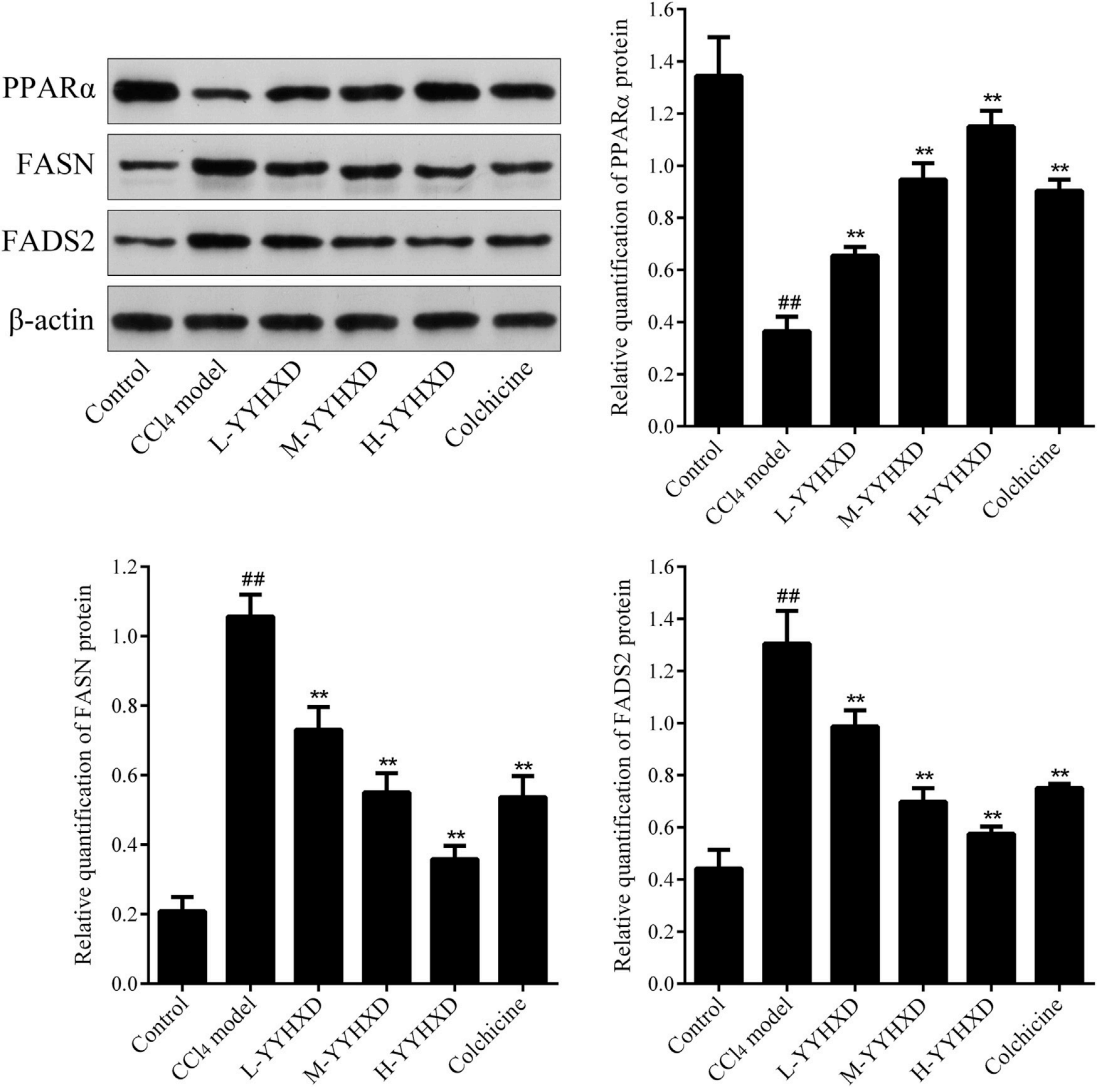
Effect of YYHXD on expression of FASN, FADS2, and PPARα proteins in hepatic fibrosis rats. ^##^ *p*-value <0.01, compared with the control group. **p-value <0.01, compared with the CCl_4_ model group.

To probe the effect of YYHXD on aliphatic acid synthesis and metabolism, we used Oil Red O staining to assess the degree of lipid accretion in hepatic tissue. Additionally, q-PCR and Western blot methods were employed to evaluate the expression of genes implicated in aliphatic acid synthesis and metabolism in hepatic tissue. ELISA was used to quantify TG, TC, and LDL-C in serum. Results from Oil Red O staining indicated no lipid accretion in the liver in the control group. In contrast, the CCl_4_ model group exhibited significant amounts of lipid accretion and noticeable fatty degeneration in hepatic tissue. Compared with the CCl_4_ model group, the YYHXD and positive control groups displayed a significant reduction of lipid droplets and less severe fat denaturation in the liver. In particular, the ability of YYHXD (20 g/10 mL/kg) to ameliorate hepatic lipid accretion induced by CCl_4_ was superior to that of colchicine (0.2 mg/10 mL/kg) (Figure 9). Serum biochemistry analysis showed that CCl_4_ increased levels of TG, TC, and LDL-C, all of which were reduced by varying degrees dose-dependently by YYHXD (Figure 10). *Fasn*, *Fads2*, and *Ppara* are key genes regulating aliphatic acid synthesis and metabolism (Hayashi et al., 2021; Li et al., 2020; O'Farrell et al., 2022; Pawlak et al., 2015; Yamane et al., 2022). Among them, *Fasn* and *Fads2* were the two most differentially expressed genes among the three groups according to the results of transcriptome sequencing. The q-PCR results indicated that expression of *Fasn* and *Fads2* mRNA in hepatic tissue of CCl_4_-induced hepatic fibrosis rats was notably higher than that of normal rats, while YYHXD significantly inhibited this increase in a dose-dependent manner (Figure 11). Additionally, the Western blot data indicated that expression of FASN, FADS2, and PPARα proteins in hepatic tissue of CCl_4_-induced hepatic fibrosis rats was notably higher than that in normal rats, while YYHXD dose-dependently inhibited the increases in these aliphatic acid metabolism-related proteins. In particular, the ability of high-dose YYHXD (20 g/10 mL/kg) to reduce aliphatic acid accretion was superior to that of colchicine (0.2 mg/10 mL/kg) (Figure 12).

### 3.6 YYHXD activated the AMPK signaling pathway in hepatic fibrosis rats

**FIGURE 13:**
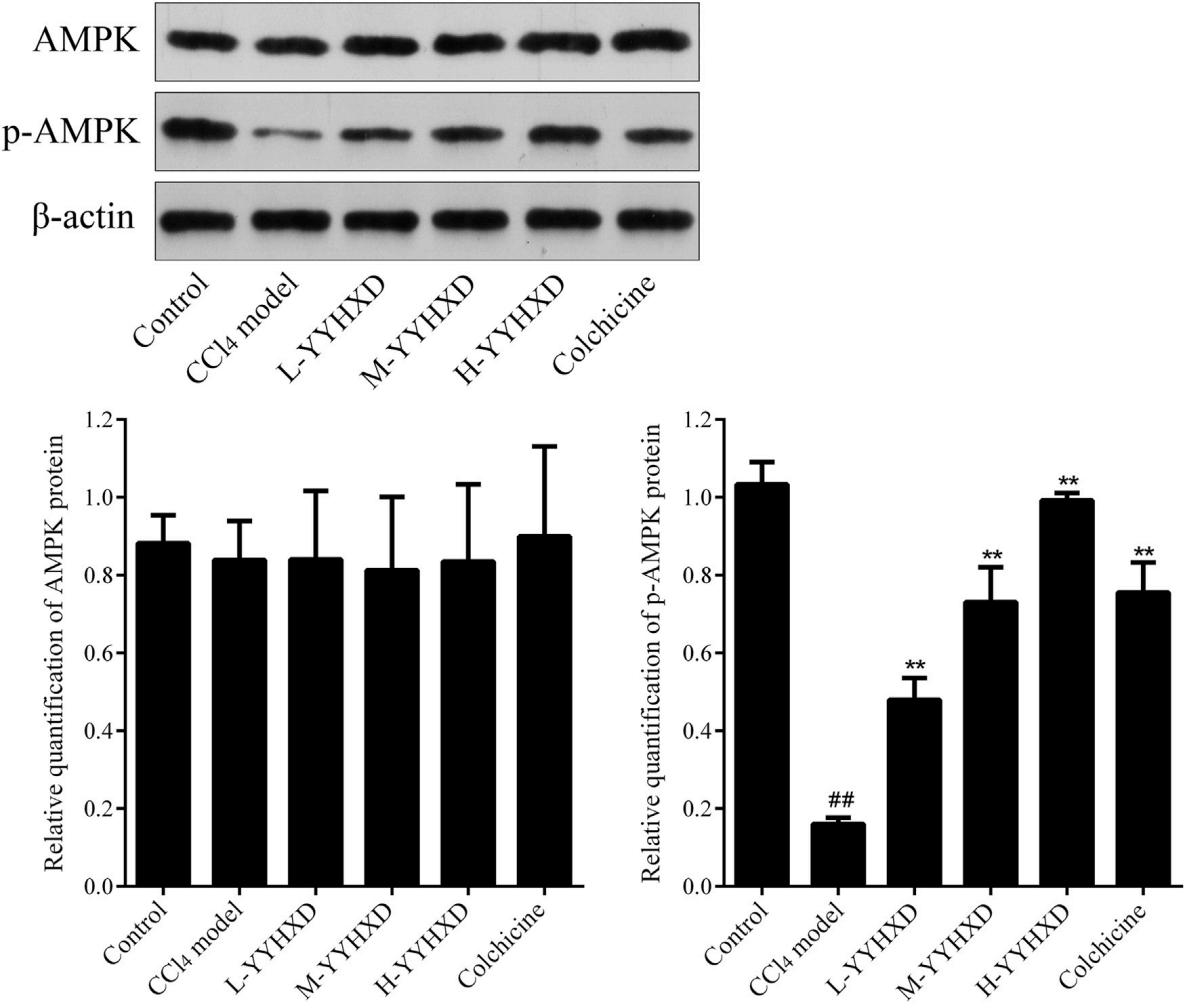
Effect of YYHXD on protein expression of AMPK and p-AMPK in hepatic fibrosis rats by Western blotting analysis. ^##^ *p*-value <0.01, compared with the control group. **p-value <0.01, compared with the CCl4 model group.

**FIGURE 14:**
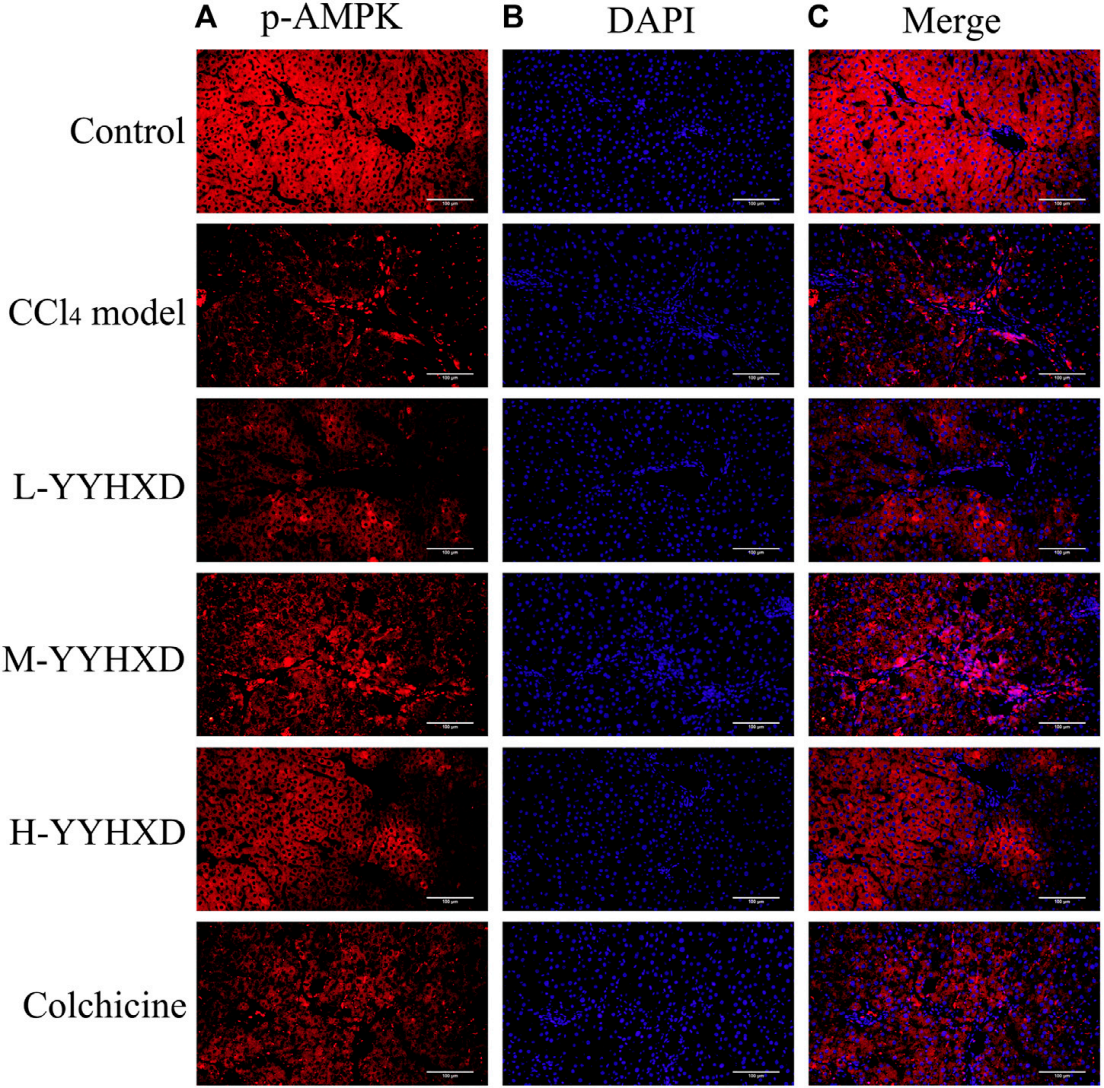
Effect of YYHXD on expression of p-AMPK in hepatic tissue of hepatic fibrosis rats by immunofluorescence staining. The panels show DAPI nuclear staining in blue **(B)**, staining with the corresponding p-AMPK antibody in red **(A)**, and merged images **(C)**. Scale: 200 µm.

To further elucidate the effect of YYHXD on AMPK signaling, Western blotting analysis was used to assess expression of AMPK and p-AMPK proteins in rat hepatic tissues from each group. Our results demonstrated that AMPK protein expression was not statistically different in each group. Compared with the control group, the levels of p-AMPK were notably lower in the hepatic fibrosis CCl_4_ model group, indicating repression of the AMPK signaling pathway. However, YYHXD therapy upregulated expression of p-AMPK, reversing the downregulation of AMPK signaling that was induced by CCl_4_ (Figure 13). In addition, immunofluorescence staining was used to confirm the levels of p-AMPK in hepatic tissue. The results of immunofluorescence staining (Figure 14) were consistent with those of the Western blot analysis. Expression of p-AMPK was notably lower in hepatic tissue of rats with hepatic fibrosis compared with that in normal rat hepatic tissue. YYHXD treatment (5, 10, and 20 g/10 mL/kg) dose-dependently attenuated the significant reduction of p-AMPK expression induced by CCl_4_ in rat hepatic tissue. Furthermore, the ability of high-dose YYHXD (20 g/10 mL/kg) to upregulate p-AMPK expression was superior to that of the positive control drug colchicine (0.2 mg/10 mL/kg).

## 4 Discussion

Hepatic fibrosis is a hepatopathy that is characterized by chronic inflammation and incremental accretion of collagen, which can advance to hepatic cirrhosis and even hepatoma. The pathogenesis of hepatic fibrosis is complex, often involving liver dysfunction, immune injury, and oxidative stress damage (Rodríguez et al., 2021). YYHXD is a traditional Chinese medicine comprising 14 botanical drugs, developed based on traditional Chinese medicine theory. Danggui and Dihuang are the principal botanical drugs, with functions that include nourishing blood, promoting blood circulation, replenishing yin, and tonifying essence. They have been proven to activate the immune regulation system (Wu et al., 2011), reduce hepatocyte death (Liu et al., 2012), maintain ECM balance, and boost regeneration of hepatocytes and blood (Wang and Liang, 2010). Baishao, Chishao, Chuanxiong, Danshen, Zhebeimu, and Zelan are regarded as the ministerial botanical drugs in the formula, which function to nourish yin, cool blood, and promote liver circulation to dispel blood stasis. Among them, Chishao can stop bleeding and disperse stasis, and is particularly suitable for treating yin deficiency with heat stasis. Danshen, Zhebeimu, and Zelan boost blood circulation and resolve blood stasis, and have been shown to play an important role in regulating the immune system (Peng et al., 2018), protecting the liver from damage (Nagappan et al., 2019; Wu et al., 2022), and impeding the advancement of liver cancer (Zhang Q. et al., 2022). Chuanxiong boosts blood circulation and relieves stagnation, while Baishao nourishes blood and liver. Both Chuanxiong and Baishao have been shown to impede the activation of hepatic stellate cells and the inflammatory response in liver (Kong et al., 2013; Wu et al., 2015). Moreover, Chaihu, Chenpi, Dahuang, Huangqin, Gancao, and Dazao are the auxiliary botanical drugs in the formula. Traditional Chinese medicine theory holds that Chaihu modulates liver function and relieves depression, Chenpi regulates qi and relieves pain, Dahuang clears heat and detoxifies, Huangqin purges fire and detoxifies, and Gancao harmonizes the properties of the other botanical drugs. Modern pharmacological studies have demonstrated that Chaihu and Chenpi ameliorated hepatic fibrosis through anti-inflammatory, antioxidant, and anti-apoptotic mechanisms (Zou et al., 2018; Wu et al., 2021). Dahuang extract emodin has been discovered to reduce liver oxidative stress injury by activating the AMP-activated protein kinase (AMPK) signaling pathway (Lee D. et al., 2020), while Huangqin extract baicalin alleviates hepatic fibrosis by inducing hepatic stellate cell ferroptosis (Liu et al., 2022). Dazao has been shown to have liver-protective, anti-cancer, antioxidant, and anti-inflammatory activities (Sun et al., 2022), and Gancao alleviates hepatic tissue inflammation and fibrosis damage (Guo et al., 2023). The combination of these botanical drugs can exert synergistic effects across many traditional therapeutic actions and pharmacological mechanisms. Therefore, YYHXD exerts yongyinghuoxue, promoting qi flow and resolving stasis effects according to TCM theory. And YYHXD has been broadly applied in China to treat various kinds of hepatopathy caused by yin deficiency with blood stasis, qi stagnation, and blood stasis, such as hepatic fibrosis, and has shown promising therapeutic effects. However, the pharmacological mechanism of YYHXD in the treatment of hepatic fibrosis is complex and not well understood, due to its multi-component nature, having effects on multiple targets and pathways. Transcriptomic techniques, with their high-throughput capabilities, have pivotal roles in elucidating the pathogenesis of hepatic fibrosis and the pharmacological mechanisms of traditional Chinese medicines with such complex therapeutic characteristics.

The CCl_4_-induced rat hepatic fibrosis model used in this study is a widely accepted and well-established animal model that recapitulates many pathological features of human hepatic fibrosis, including excessive deposition of extracellular matrix, activation of hepatic stellate cells, and development of cirrhosis (O’Farrell et al., 2022; Guo et al., 2023). While there are some differences between the rat model and human disease, such as the etiology and the time course of fibrosis development, the fundamental pathogenic mechanisms are highly similar. Previous studies have shown that the therapeutic effects of traditional Chinese medicines, including YYHXD, observed in the CCl4-induced rat model often translate well to human hepatic fibrosis patients (Ma et al., 2018; Liang et al., 2022). Therefore, the findings from the present study using the CCl4-induced rat model provide valuable insights into the potential pharmacological mechanisms of YYHXD in the treatment of human hepatic fibrosis.

In this work, transcriptomic technology was utilized to identify potential target genes in YYHXD therapy of hepatic fibrosis. Using bioinformatics analysis, the biological functions and signaling pathways associated with these target genes were identified. Altogether 96 genes implicated in YYHXD therapy for hepatic fibrosis were identified from RNA sequencing data, with *Fasn* and *Fads2* being potential key genes affected by treatment. Functional enrichment analysis revealed that YYHXD may exert its therapeutic effects on hepatic fibrosis through regulation of important pathways such as aliphatic acid biosynthesis and metabolism, as well as the AMPK signaling pathway.

Aliphatic acid synthesis and metabolic pathways are crucial players in the occurrence and advancement of hepatic fibrosis. As a major organ for aliphatic acid synthesis and metabolism, the liver has the ability to synthesize, break down, and metabolize aliphatic acids (Heeren and Scheja, 2021). In certain pathological states, such as obesity, hyperlipidemia, and hepatic fibrosis, the liver is susceptible to excessive lipid accretion (Ran et al., 2023). Adipocyte proliferation, white blood cell infiltration, and cytokine release can induce the deposition and accretion of lipids in hepatocytes, exacerbating the occurrence and progression of hepatic fibrosis (Suganami et al., 2012; van der Windt et al., 2018; Heeren and Scheja, 2021). Transforming growth factor-beta (TGF-β), a critical mediator in hepatic fibrosis, is able to stimulate hepatic stellate cell diffusion as well as collagen deposition. Overexpression of TGF-β impedes the expression of genes implicated in aliphatic acid metabolism and the activity of enzymes, leading to abnormal aliphatic acid metabolism and excessive lipid accretion, which exacerbates the occurrence and progression of hepatic fibrosis (Kwapisz et al., 2021). Abnormalities in aliphatic acid synthesis and metabolism also cause oxidative stress and inflammatory responses, accelerating the development of hepatic fibrosis (Zhang et al., 2023). FASN, FADS2 and PPARα are important regulators of aliphatic acid synthesis and metabolism pathways. FASN boosts aliphatic acid synthesis and is involved in various metabolic processes in hepatocytes, and its expression is positively correlated with the severity of hepatic fibrosis (O'Farrell et al., 2022). FADS2 is a pivotal enzyme during aliphatic acid desaturation, which is essential for biosynthesis of long-chain polyunsaturated aliphatic acids (PUFAs) (Yamane et al., 2022). Increased expression of FADS2 significantly augments the development of hepatic fibrosis, causing accelerated synthesis of unsaturated aliphatic acids in hepatocytes and an imbalance of intracellular lipid metabolism (Li et al., 2020; Hayashi et al., 2021). PPARα is another essential regulator of aliphatic acid metabolism. Inhibition of PPARα can lead to lipid metabolism disorder and promote the progression of hepatic fibrosis. Activation of PPARα can accelerate the breakdown of aliphatic acids and inhibit activation of hepatic stellate cells, reducing the severity of hepatic fibrosis (Pawlak et al., 2015).

AMPK is an important intracellular metabolic regulator that can affect aliphatic acid synthesis and metabolism by modulating various signaling pathways. This makes it a key player in the occurrence and progression of hepatic fibrosis. Activation of AMPK can inhibit aliphatic acid synthesis and boost aliphatic acid oxidation, resulting in reduced accretion of liver fat and progression of hepatic fibrosis (Zhao and Saltiel, 2020; Hu et al., 2021). Activated AMPK (p-AMPK) can reduce the synthesis of acyl-CoA, which has a key role in aliphatic acid synthesis, and inhibits aliphatic acid synthesis, leading to reduced fat deposition (Ruderman et al., 2003). Moreover, Viollet et al. (2009) suggested that activation of AMPK can also promote β-oxidation of aliphatic acids in the liver, increase the rate of aliphatic acid metabolism, and alleviate lipid deposition. In addition to directly regulating aliphatic acid metabolism, AMPK activation can also exert regulatory effects through related genes and transcription factors. For example, AMPK activation can inhibit expression of sterol regulatory element-binding protein 1c (SREBP-1c), reducing the expression of genes implicated in aliphatic acid synthesis (Yap et al., 2011). AMPK activation can also regulate aliphatic acid metabolism by modulating the expression of cytochrome P450 enzyme 2E1 (CYP2E1) (Ki et al., 2007; Kim et al., 2016). According to relevant research, p-AMPK can also alleviate hepatic fibrosis by suppressing the release of inflammatory mediators and collagen synthesis (Shi et al., 2017; Huang et al., 2021). AMPK can downregulate expression of FASN, impede hepatic intracellular aliphatic acid synthesis and saturation, and ameliorate fatty liver disease (Lee E. et al., 2020; Irungbam et al., 2020). Furthermore, AMPK activation is able to enhance aliphatic acid β-oxidation by activating PPARα, suppressing cell diffusion, inflammation, apoptosis, and other cellular processes (Sozio et al., 2011; Mansour et al., 2022).

In this work, transcriptomic sequencing and experimental verification were used to deconvolute the pharmacological mechanisms underlying the efficacy of YYHXD in therapy of hepatic fibrosis injury in rats. It was found that efficacy correlated with the modulation of aliphatic acid synthesis and metabolism pathways, as well as the AMPK signaling pathway. The observed dose-dependent therapeutic effects of YYHXD provide valuable insights into its pharmacodynamics. The superior efficacy of the high-dose YYHXD suggests that a more comprehensive modulation of the multiple pathways involved in hepatic fibrosis, such as aliphatic acid metabolism and the AMPK signaling pathway, may be achieved with higher concentrations of the decoction’s phytochemical constituents. This dose-dependent effect highlights the importance of optimizing the dosage regimen to maximize the therapeutic potential of YYHXD in the clinical management of hepatic fibrosis. This finding was novel, although some botanical drugs used in YYHXD have been found to regulate aliphatic acid synthesis and metabolism or activate the AMPK signaling pathway. For example, Qiang Zhang et al. found that the active metabolite of Radix Bupleuri, β-sitosterol, reduced lipid accretion in hepatocytes processing free aliphatic acids by activating the PPARγ-UCP-1 pathway, thus exerting anti-hepatic fibrosis effects (Zhang Y. et al., 2022). Lei et al. (2022) found that the traditional Chinese medicine formula Chaihu Shugan powder (containing Radix Bupleuri as the main metabolite) ameliorated lipid accretion in the livers of mice with non-alcoholic fatty liver disease, inhibited secretion of inflammatory cytokines in hepatic tissues, and inhibited synthesis of aliphatic acids in the liver. The active metabolite of Salvia miltiorrhiza, cryptotanshinone, activates the AMPK signaling pathway, inhibits fat formation, aliphatic acid oxidation, oxidative stress, and inflammatory reactions, thus ameliorating ethanol-induced liver damage (Nagappan et al., 2019).

However, several methodological limitations and uncertainties should be acknowledged. First, the small sample size for the RNA-seq analysis may limit the statistical power, and the use of whole liver tissue precludes the identification of cell-type specific effects. Second, the CCl4-induced rat model may not fully recapitulate the complex etiology and pathogenesis of human liver fibrosis, and the findings need to be validated in additional animal models. Lastly, this study focused on transcriptional changes, and further exploration of post-transcriptional and post-translational regulation would provide a more comprehensive understanding of the mechanisms. Despite these limitations, the results suggest that YYHXD exerts anti-fibrotic effects through the modulation of aliphatic acid synthesis/metabolism and the AMPK signaling pathway, which have potential clinical relevance for the management of liver fibrosis and related liver diseases. Future research directions include evaluating YYHXD in additional animal models, exploring its combination with other therapies, conducting human clinical trials, and deeper mechanistic investigations to optimize its therapeutic applications.

## 5 Conclusion

In this work, the pharmacological mechanisms of YYHXD *in vivo* were investigated for the first time. Our results demonstrated that YYHXD effectively reversed CCl_4_-induced hepatic fibrosis in rats, with corresponding restoration of liver function, reduced oxidative stress, and alleviated inflammatory damage. The anti-hepatic fibrosis effect of YYHXD was characterized by modulation of aliphatic acid synthesis and metabolism, combined with activation of the AMPK signaling pathway. This work provided a reference and foundation for further research and clinical utilization of YYHXD.

## Data Availability

The datasets presented in this study can be found in online repositories. The names of the repository and accession number(s) can be found below: NCBI Sequence Read Archive (SRA), accession number PRJNA1059506.
